# A framework for assessing 16S rRNA marker-gene survey data analysis methods using mixtures.

**DOI:** 10.1186/s40168-020-00812-1

**Published:** 2020-03-13

**Authors:** Nathan D. Olson, M. Senthil Kumar, Shan Li, Domenick J. Braccia, Stephanie Hao, Winston Timp, Marc L. Salit, O. Colin Stine, Hector Corrada Bravo

**Affiliations:** 1 Biosystems and Biomaterials Division, National Institute of Standards and Technology, 100 Bureau Dr., Gaithersburg, 20899 MD USA; 2 Center for Bioinformatics and Computational Biology, University of Maryland, College Park, 8314 Paint Branch Dr., College Park, 20742 MD USA; 3 University of Maryland Institute of Advanced Computer Studies, University of Maryland, College Park, 8223 Paint Branch Dr., College Park, 20742 MD USA; 4 Department of Epidemiology and Public Health, University of Maryland School of Medicine, 660 W. Redwood St., Baltimore, 21201 MD USA; 5 Department of Biomedical Engineering, Johns Hopkins University, 720 Rutland Ave., Baltimore, 21205 MD USA; 6 Joint Initiative for Metrology in Biology, 443 Via Ortega, Stanford, 94305 CA USA; 7 Department of Computer Science, University of Maryland, College Park, 8223 Paint Branch Dr., College Park, 20742 MD USA

**Keywords:** 16S rRNA gene, Assessment, Bioinformatic pipeline, Normalization, Differential abundance

## Abstract

**Background:**

There are a variety of bioinformatic pipelines and downstream analysis methods for analyzing 16S rRNA marker-gene surveys. However, appropriate assessment datasets and metrics are needed as there is limited guidance to decide between available analysis methods. Mixtures of environmental samples are useful for assessing analysis methods as one can evaluate methods based on calculated expected values using unmixed sample measurements and the mixture design. Previous studies have used mixtures of environmental samples to assess other sequencing methods such as RNAseq. But no studies have used mixtures of environmental to assess 16S rRNA sequencing.

**Results:**

We developed a framework for assessing 16S rRNA sequencing analysis methods which utilizes a novel two-sample titration mixture dataset and metrics to evaluate qualitative and quantitative characteristics of count tables. Our qualitative assessment evaluates feature presence/absence exploiting features only present in unmixed samples or titrations by testing if random sampling can account for their observed relative abundance. Our quantitative assessment evaluates feature relative and differential abundance by comparing observed and expected values. We demonstrated the framework by evaluating count tables generated with three commonly used bioinformatic pipelines: (i) DADA2 a sequence inference method, (ii) Mothur a de novo clustering method, and (iii) QIIME an open-reference clustering method. The qualitative assessment results indicated that the majority of Mothur and QIIME features only present in unmixed samples or titrations were accounted for by random sampling alone, but this was not the case for DADA2 features. Combined with count table sparsity (proportion of zero-valued cells in a count table), these results indicate DADA2 has a higher false-negative rate whereas Mothur and QIIME have higher false-positive rates. The quantitative assessment results indicated the observed relative abundance and differential abundance values were consistent with expected values for all three pipelines.

**Conclusions:**

We developed a novel framework for assessing 16S rRNA marker-gene survey methods and demonstrated the framework by evaluating count tables generated with three bioinformatic pipelines. This framework is a valuable community resource for assessing 16S rRNA marker-gene survey bioinformatic methods and will help scientists identify appropriate analysis methods for their marker-gene surveys.

## Background

Studies often use 16S rRNA marker-gene surveys (targeted sequencing of the 16S rRNA gene) to characterize microbial communities. The 16S rRNA marker-gene-survey measurement process includes molecular steps to selectively target and sequence the 16S rRNA gene, and computational steps to convert the raw sequence data into a count table of feature relative abundance values [1]. Both molecular and computational measurement processes contribute to the overall measurement bias and dispersion [1–3]. Datasets that characterize complex microbial communities with some degree of “ground truth” are needed to properly characterize the 16S rRNA marker-gene-survey measurement process accuracy.

Diverse bioinformatic pipelines used in practice produce count tables with diverse characteristics. For example, the commonly used QIIME, Mothur, and DADA2 pipelines produce feature sets and count tables with different characteristics. The QIIME open-reference clustering pipeline performs feature inference using reference clusters and full amplicon pairwise distances [4, 5]. The Mothur de novo clustering pipeline performs feature inference using pairwise distances calculated from informative positions of a multiple sequence alignment [6, 7]. Unlike QIIME, the Mothur feature representative sequences are not the full amplicon due to the removal of informative positions after the multiple sequence alignment. The DADA2 pipeline performs feature inference using a probability model and an expectation maximization algorithm [8]. Unlike distance-based clustering methods employed by the Mothur and QIIME pipelines, DADA2 parameters determine if low abundance sequences are grouped with a higher abundance sequences.

Numerous studies have evaluated qualitative and quantitative characteristics of the rRNA measurement process using mock communities, simulated data, and environmental samples. Assessments based on mock communities [9] show features sets that commonly contain significantly more features than expected [10]. The higher than expected number of features is often attributed to sequencing and PCR artifacts as well as reagent contaminants [3, 11]. A notable exception is count tables generated using feature inference methods, such as DADA2 [8]. Sequence inference methods aim to reduce the number of features from sequence artifacts by using statistical models to group sequences by both similarity and abundance. Nonetheless, while mock communities are useful in this type of assessment, they lack the diversity and dynamic range of features present in real samples [9].

Quantitative assessment of 16S rRNA sequencing using mock communities and simulated data provides an incomplete characterization of the measurement process. Results from relative abundance estimates using mock communities generated from mixtures of single organisms’ DNA have shown taxonomic-specific effects where individual taxa are under- or over-represented in a sample. For example, Gram-negative bacteria have higher extraction efficiency compared to Gram-positive bacteria and are thus likely over-represented in count tables [12, 13]. Mismatches in the primer binding sites are also responsible for taxonomic-specific biases [3, 14, 15]. Additionally, taxon-specific biases due to sequence template properties such as GC content, secondary structure, and gene flanking regions have been observed [15–17]. Due to limited community complexity, the applicability of mock community assessment results to more complex environmental samples is unknown. Environmental sample complexity can be modeled using simulations. For example, simulations have been used to assess differential abundance methods, where specific taxa are artificially over-represented in one set of samples compared to another [18]. However, simulated data can only evaluate the computational steps of the measurement process.

Quantitative and qualitative assessment can also be performed using sequence data generated from mixtures of environmental samples. While simulated data and mock communities are useful in evaluating and benchmarking new methods, one needs to consider that methods optimized with mock communities and simulated data are not necessarily optimized for the sequencing error profile and feature diversity of real environmental samples. Data from real environmental samples are often used to benchmark new molecular laboratory and computational methods. However, without expected values for use in assessment, only measurement precision or agreement with other methods can be evaluated. By mixing environmental samples, expected values are calculated using information from the unmixed samples and the mixture design. Mixtures of environmental samples were previously used to evaluate gene expression measurements [19–21].

Here, we present a framework for assessing 16S rRNA marker-gene-survey computational analysis methods. The framework includes a 16S rRNA two-sample titration dataset (generated using mixtures of human stool sample DNA extracts) and metrics for assessing count table quantitative and qualitative characteristics. We demonstrated the framework by comparing count tables generated using three commonly used bioinformatic pipelines. Both the dataset and metrics developed in this study are publicly available and can be used to evaluate and optimize new and existing bioinformatic pipelines.

## Results

### Assessment framework

**Fig. 1:**
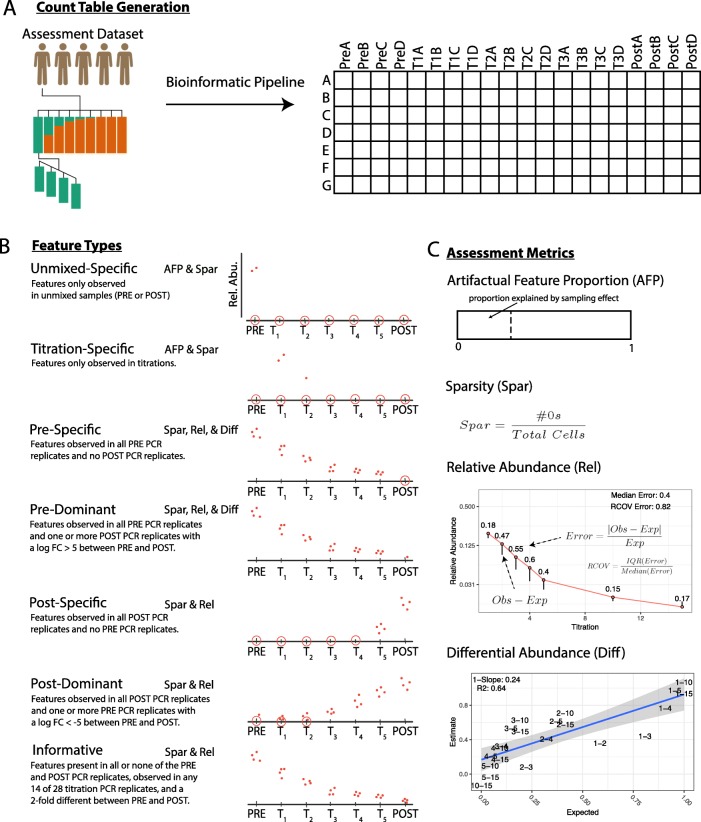
Assessment framework. **a** Count tables evaluated by the assessment framework are generated from the assessment dataset using marker-gene survey bioinformatic pipelines. Count table rows are features identified by the bioinformatic pipeline and columns are samples, four PCR replicates (labeled A–D) were sampled for PRE and POST and titrations, to simplify the diagram only three titrations are shown(labeled T1–T3). **b** The pictorial depiction and description of the seven feature types used in the assessment framework. **c** Qualitative and quantitative assessment metrics used in the assessment framework. Artifactual feature proportion (AFP) and Sparsity (SPAR) were used in the qualitative assessment. The artifactual feature proportion metric (AFP) is a qualitative assessment of feature presence/absence based on unmixed-specific or titration-specific artifactual features. Sparsity (SPAR) is a qualitative assessment of the proportion of observed features in each sample relative to the total observed features. For the quantitative assessment relative (Rel) and differential abundance (Diff), bias and variance metrics were used. For the quantitative assessment metrics, the relative abundance (Rel) and differential abundance (Diff) plots of example features are used to describe how the bias and variance metrics are calculated. For the relative abundance (Rel) metrics the error rate (|Obs-Exp|/Exp) is calculated for individual titrations and the bias (median(error)) and variance (RCOV) metrics are summaries the error rates by feature. For the differential abundance (Diff) plot points represent the log fold-change between two titrations, point text indicates the titrations compared. A linear model is fit to the data and the model fit information is used for the differential abundance bias (|1−slope|) and variance metrics (*R*^2^). Each feature type in **b** is labeled with the assessments shown in **c** in which they are employed

Our framework assesses the qualitative and quantitative characteristics of the 16S rRNA measurement process (Fig. 1). The framework evaluates count tables generated by bioinformatic pipelines from a dataset developed specifically for use in this framework. The qualitative assessment provides insight into how much confidence a user can have in feature presence/absence. The quantitative assessment evaluates the bias and variance of relative and differential abundance estimates.

#### Assessment dataset—mixture design

**Fig. 2:**
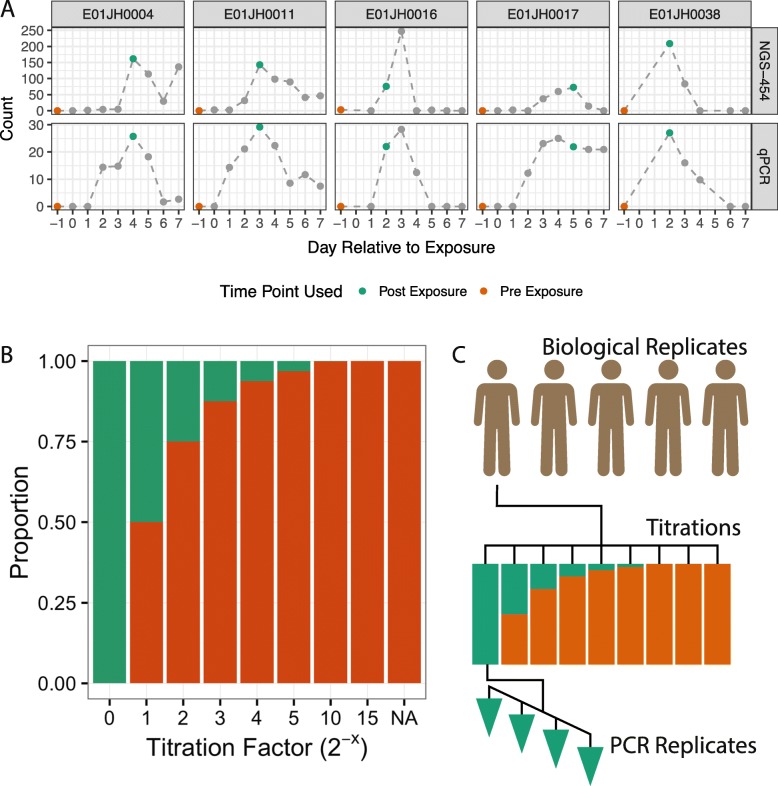
Sample selection and experimental design for the two-sample titration 16S rRNA marker-gene-survey assessment dataset. **a** Pre- and post-exposure (PRE and POST) samples from five vaccine trial participants were selected based on *Escherichia coli* abundance measured using qPCR and 454 16S rRNA sequencing (454-NGS), data from Pop et al. [22]. Counts represent normalized relative abundance values for 454-NGS and copies of the heat-labile toxin gene per microliter, a marker gene for ETEC, for qPCR. PRE and POST samples are indicated with orange and green data points, respectively. Gray points are other samples from the vaccine trial time series. **b** Proportion of DNA from PRE and POST samples in titration series samples. PRE samples were titrated into POST samples following a log_2_ dilution series. The NA titration factor represents the unmixed PRE sample. **c** PRE and POST samples from the five vaccine trial participants, subjects, were used to generate independent two-sample titration series. Four replicate PCRs were performed for each of the 45 samples, 7 titrations + 2 unmixed samples times 5 subjects, resulting in 190 PCRs

Using mixtures of environmental samples, we generated a dataset with expected values for our assessment framework. For mixture datasets, expected values can be obtained using information from unmixed samples and the mixture design. Our mixture dataset uses a two-sample titration mixture design, where DNA collected from five vaccine trial participants before and after exposure to pathogenic *Escherichia coli* was mixed following a log_2_ dilution series (Fig. 2). Each sample was sequenced in quadruplicate. For our two-sample titration mixture design, expected feature relative abundance is calculated using Eq. 1, where *θ*_*i*_ is the proportion of POST DNA in titration *i*, *q*_*ij*_ is the relative abundance of feature *j* in titration *i*, and the relative abundance of feature *j* in the unmixed PRE and POST samples is *q*_pre,*j*_ and *q*_post,*j*_. Throughout the rest of the manuscript, samples collected prior to and after *E. coli* exposure are referred to as PRE and POST, respectively.

1 $$ q_{ij} = \theta_{i} q_{{\text{post}},j} + (1 - \theta_{i}) q_{{\text{pre}},j}  $$

#### Qualitative assessment

The qualitative assessment shows how well pipelines differentiate true biological sequences from measurement process artifacts. Inadequate processing of artifacts results in false-positive and false-negative features where false-positives are features in a count table not in the sequenced sample, and false-negatives are biological sequences in a sample not represented in the count table. Our qualitative assessment methods characterize the artifactual feature proportion (the frequency of artifactual features in a count table) by estimating the proportion of *titration-* and *unmixed-specific* features (Fig. 1b) that cannot be accounted for by sampling alone. We combine the artifactual feature proportion assessment results with sparsity estimates to hypothesize whether the artifactual features are primarily false-positives or false-negatives. Sparsity is defined as the fraction of 0 valued cells in the count table (Fig. 1c).

#### Quantitative assessment

To evaluate count table abundance values, our quantitative assessment uses error, bias, and variance metrics (Fig. 1c). Error metrics measure agreement between observed and expected abundance values. The bias and variance metrics summarize feature-level performance. Bias metrics summarize the overall agreement with expected values and the variance metric characterizes the distribution of the agreement. Overall, pipeline performance is evaluated by comparing count table metric distributions. Additionally, feature-level metrics are indicators of feature-specific biases.

### Assessment dataset characterization and validation

To assure the mixture dataset is suitable for our assessment framework, we first validated the titration series and raw sequence data. The mixture dataset had sufficient sample coverage, reads per sample, and read quality for use in our assessment framework. The number of reads per sample and distribution of base quality scores by position was consistent across individuals (Fig. S5). There were 8.9548×10^4^ (152,267–3195) sequences per sample, median and range. Average base quality score was greater than 30 over the length of the amplicon when considering both forward and reverse reads (Fig. S5B).

**Fig. 3:**
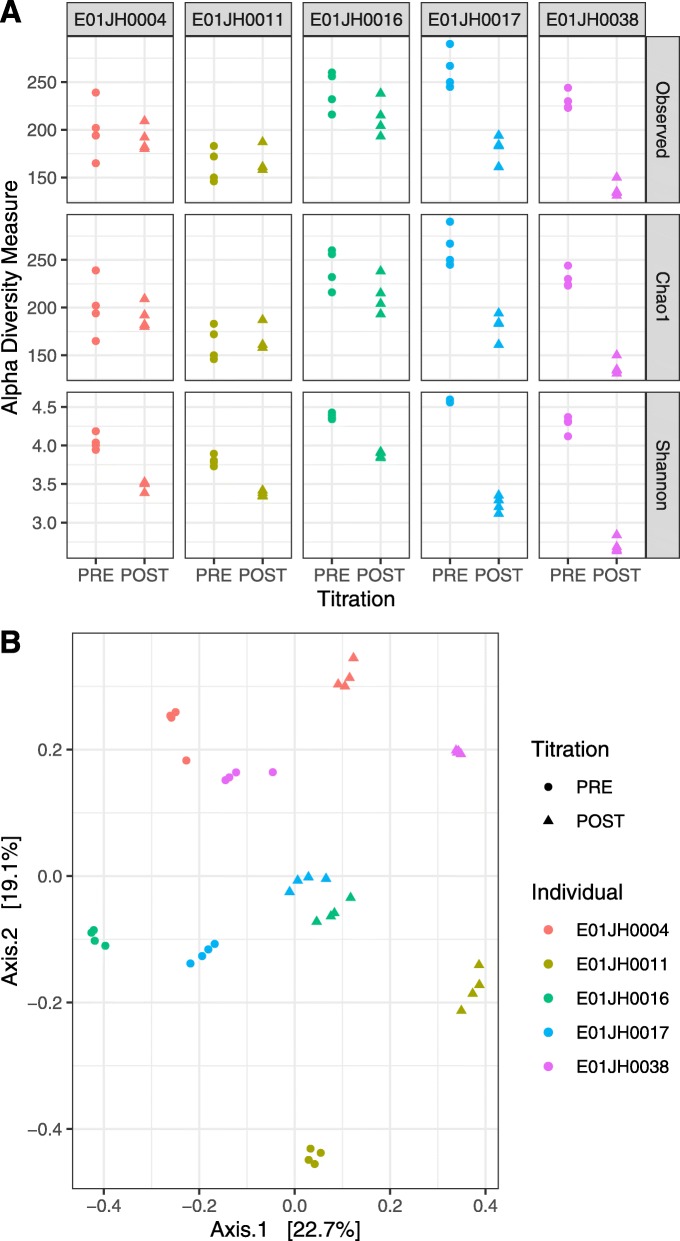
Diversity metrics for PRE and POST samples by individual. *α* (**a**) and *β* (**b**) diversity was calculated using the DADA2 count table. Beta diversity was calculated using Bray-Curtis diversity metric, and principal components analysis was used for ordination. The same color and shape scales were used for plots A and B

Additionally, we characterized individual-specific differences to inform the interpretation of our assessment results. No subject-specific differences in base quality score were observed (Fig. S5). However, average read depth was greater for E01JH004 compared to the other individuals (Fig. S5). Community composition differences between PRE and POST samples and individuals were characterized using alpha and beta diversity (Fig. 3). Overall alpha diversity was higher for PRE except for E01JH0011, though differences in diversity between PRE and POST varied by individual. Based on the beta diversity the community composition within individuals differed between the PRE and POST samples. Note that the assessment metrics defined above and results below are based on within individual comparisons.

To validate the two-sample titration assessment dataset, we evaluated two assumptions about the titrations: (1) the samples were mixed volumetrically in a log_2_ dilution series according to the mixture design. (2) The unmixed PRE and POST samples have the same proportion of prokaryotic DNA. To validate the sample volumetric mixing exogenous DNA (ERCC plasmids) were spiked into the unmixed samples before mixing and quantified using qPCR (Fig. S1B). The stool samples used to generate the mixtures have both eukaryotic (primarily human) DNA and prokaryotic DNA. If the proportion of prokaryotic DNA differs between the unmixed samples, then the amount of DNA from the unmixed samples in a titration targeted by 16S rRNA gene sequencing is not consistent with the mixture design. We quantified the proportion of prokaryotic DNA in the unmixed samples using a qPCR assay targeting the 16S rRNA gene (Fig. S1C).

Our assessment dataset validation results indicated that the samples were volumetrically mixed according to the mixture design (Table S1) but prokaryotic DNA proportion varied across the titration series (Fig. S2). To account for deviations from the mixture design due to differences in the proportion of prokaryotic DNA in the unmixed samples, we estimated the proportion of POST in each titration using the 16S rRNA sequencing data (Fig. S3), and the estimated POST proportions were used in our assessment metric calculations. See for the assessment dataset validation methods and results.

**Count table assessment demonstration** Next, we demonstrate the utility of our assessment framework on count tables generated using three different bioinformatic pipelines; DADA2, Mothur, and QIIME. First, we provide high level summary statistics for initial insight into how the count tables differ. Next, we compare the assessment framework results for the three count tables. We summarize the pipeline assessment results in Table 2.

**Count table characteristics**

The count tables generated using the three bioinformatic pipelines vary in pre-processing and feature inference methods. These differences are reflected in the number of features, total abundance, and drop-out rate (Table 1, Fig. S6B). The pipelines evaluated employ different approaches for handling low quality reads resulting in large differences in the drop-out rate, that is, the fraction of raw sequences not included in the count table (Table 1). QIIME pipeline has the highest drop-out rate and number of features per sample but fewer total features than Mothur (Fig. S6). The targeted amplicon region has a relatively small overlap region, 136 bp for 300 bp paired-end reads, compared to other commonly used amplicons [23, 24]. The high drop-out rate is due to low basecall accuracy at the ends of the reads especially the reverse reads resulting in a high proportion of unsuccessfully merged reads pairs (Fig. S5B). Further increasing the drop-out rate, QIIME excludes singletons (features only observed once in the dataset).

**Table 1:** Summary statistics for the different bioinformatic pipelines

Pipelines	Features	Sparsity	Total abundance	Drop-out rate
DADA2	3 144	0.93	68 649 (1661–112 058)	0.24 (0.18–0.59)
Mothur	3 8358	0.98	53 775 (1265–87 806)	0.40 (0.35–0.62)
QIIME	11 385	0.94	25 254 (517–46 897)	0.70 (0.62–0.97)

DADA2 is a denoising sequence inference pipeline, QIIME is an open-reference clustering pipeline, and Mothur is a de novo clustering pipeline. NTC samples (no template controls) were excluded from summary statistics. Sparsity is the proportion of 0’s in the count table. Features is the total number of OTUs (QIIME and Mothur) or SVs (DADA2) in the count tables. Sample coverage is the median and range (minimum-maximum) per sample total abundance. Drop-out rate is the proportion of reads removed while processing the sequencing data for each bioinformatic pipeline

**Table 2:** Count table assessment summary

Assessment	Measurand	Metric	Pipelines	Rank
Qualitative	Presence/ absence	AFP	DADA2	3
			Mothur	1
			QIIME	1
		Sparsity	DADA2	1
			Mothur	3
			QIIME	2
Quantitative	Relative abundance	Bias	DADA2	1
			Mothur	2
			QIIME	3
		Variance	DADA2	1
			Mothur	1
			QIIME	1
	Differential abundance	Bias	DADA2	3
			Mothur	1
			QIIME	1
		Variance	DADA2	1
			Mothur	1
			QIIME	1

Pipelines are ranked (1–3) based on performance for each component of the assessment framework

Feature taxonomic composition also varied by pipeline (Fig. S7). The three pipelines generated unique feature sets in terms of sequence length and amplicon position (see pipeline description). Therefore, we used feature taxonomic assignments for cross-pipeline community composition comparison. Phylum and order relative abundances are similar across pipelines (Fig. S7A and B). We attribute the observed differences to different taxonomic classification methods and databases used by the pipelines. Regardless of the relative abundance threshold, most genera were unique to individual pipelines (Fig. S7C and D). QIIME shared the fewest genera with the other pipeline. QIIME was the only pipeline to use open-reference clustering and the Greengenes database. Mothur and DADA2 both used the SILVA dataset. The Mothur and DADA2 pipeline use different implementations of the RDP naïve Bayesian classifier, which may be partially responsible for the Mothur, unclustered, and DADA2 differences.

#### Qualitative assessment

**Fig. 4:**
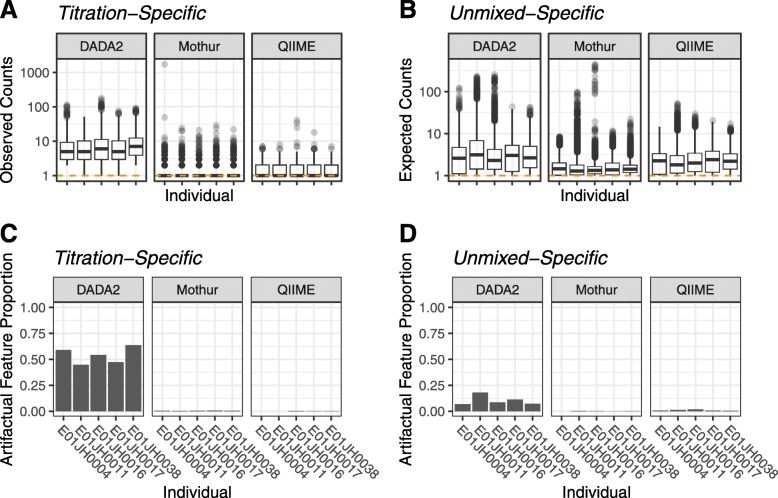
Distribution of **a** observed count values for *titration-specific* (TS) features and **b** expected count values for *unmixed-specific* (US) features by pipeline and individual. The orange horizontal dashed line indicates a count value of 1. **c** Artifactual feature proportion (Art. Feat. Prop.) for *titration-specific* and **d***unmixed-specific* features with an adjusted *p*value < 0.05 for the Bayesian hypothesis test and binomial test respectively. We failed to accept the null hypothesis when the *p* value < 0.05, indicating that the discrepancy between the feature only being observed in the titrations or unmixed samples cannot be explained by sampling alone

To evaluate feature presence-absence, the framework’s qualitative assessment measures artifactual feature proportion and count table sparsity. Low abundance features present only in unmixed samples or titration samples are expected due to random sampling. Unmixed- and titration-specific features were observed for all pipelines (titration-specific: Fig. 4a, unmixed-specific: Fig. 4b). Overall, the DADA2 count table had the largest number of artifactual features (Table S3).

We next assessed the proportion of these artifactual features that could be accounted for by sampling effects alone. For our two-sample titration dataset, there were unmixed-specific features with expected counts which could not be accounted for by sampling alone for all individuals and bioinformatic pipelines (Fig. 4d). However, the proportion of unmixed-specific features that could not be accounted for by sampling alone varied by bioinformatic pipeline. DADA2 had the highest proportion of unmixed-specific artifactual features whereas Mothur had the lowest proportion which is consistent with the distribution of titration-specific observed feature counts (Fig. 4c, Table S3). Based on the titration-specific artifactual features taxonomic assignments, the features are unlikely contaminants as no genera were consistently observed across pipeline and individual (Fig. S8 and S9).

We expected this mixture dataset to be less sparse relative to other datasets due to the redundant nature of the samples where the 35 titration samples are derived directly from the 10 unmixed samples, along with 4 PCR replicates for each sample. We observed overall sparsity of 0.93 and 0.94 for DADA2 and QIIME, respectively, and a higher value of 0.98 for Mothur (Table 1).

To account for differences in microbial community composition across the five individuals, we also measured sparsity at the individual level (Table S2). Individual-level sparsity is lower than overall sparsity for all three pipelines. Average sparsity across individuals was lowest for DADA2 (0.75), followed by QIIME (0.84) and Mothur (0.94).

Based on the artifactual feature proportions and count table sparsity, DADA2 artifactual features are likely due to false-negative features, whereas the Mothur and QIIME high sparsity values were attributed to false-positive features. Based on the observed sparsity levels it is unlikely that any of the pipelines successfully filtered out a majority of the sequencing artifacts. Unmixed- and titration-specific features (regardless of whether they are explained by sampling alone) contribute to sparsity and differences in the artifactual feature proportion and sparsity provide insight into how the pipelines treat sequencing artifacts.

#### Quantitative assessment

**Fig. 5:**
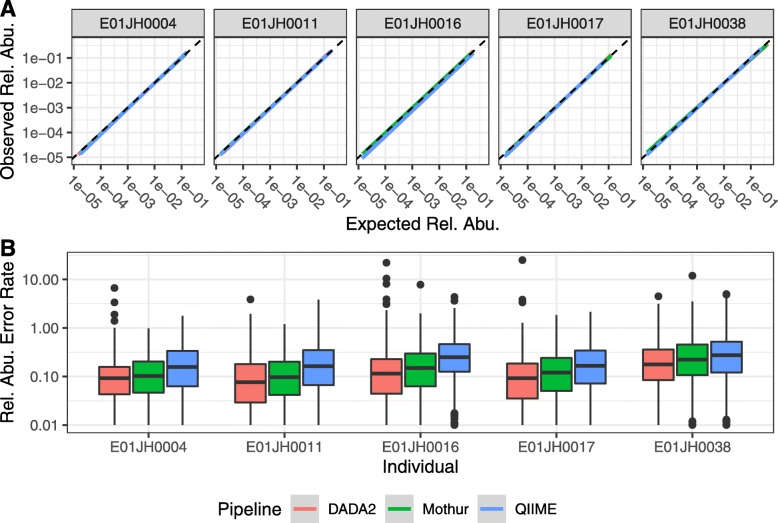
Relative abundance assessment. **a** A linear model of the relationship between the expected and observed relative abundance. The dashed gray line indicates the expected 1-to-1 relationship. The plot is split by individual and bioinformatic pipeline indicated by line color. A negative binomial model was used to calculate an average relative abundance estimate across PCR replicates. To highlight quantitative performance for higher abundance features, points with observed and expected relative abundance values less than 1/median(total abundance) were excluded from the plot. **b** Relative abundance error rate (|expected - observed|/expected) distribution by individual and pipeline

**Relative abundance assessment** To assess count table feature relative abundance values, we evaluated the consistency of the observed and expected relative abundance estimates for a feature and titration as well as feature-level bias and variance. Only features observed in all PRE and POST PCR replicates and PRE and POST specific features were included in the analysis (Table S3). Overall, agreement between inferred and observed relative abundance was high for all individuals and bioinformatic pipelines (Fig. 5a). The error rate distribution was consistent across pipelines (Fig. 5b).

**Fig. 6:**
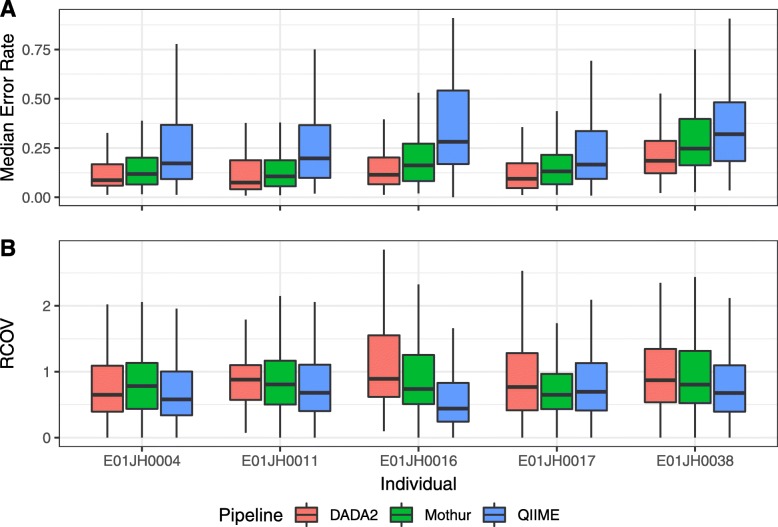
Comparison of pipeline relative abundance assessment feature-level error metrics. Distribution of feature-level relative abundance (**a**) bias metric is the median error rate and (**b**) variance is the robust coefficient of variation (RCOV=IQR/|median error rate|) by individual and pipeline. For both the bias and variance metrics, lower values are better. Boxplot outliers, 1.5×IQR from the median were excluded from the figure to prevent extreme metric values from obscuring metric value visual comparisons

To assess quantitative accuracy across pipelines, we compared the feature-level relative abundance error rate bias and variance using mixed effects models. To control for individual-specific differences, individual was included in the model as a random effect. Large variance metrics were observed for all pipelines and individuals (Table S4). The Mothur, DADA2, and QIIME feature-level biases were all significantly different from each other (*p*<1×10^−6^). DADA2 had the lowest mean feature-level bias (0.13), followed by Mothur (0.17), with QIIME having the highest bias (0.27) (Fig. 6a). The feature-level variance was not significantly different between pipelines, with QIIME having the lowest mean variance metric QIIME = 0.68, Mothur = 0.81, and DADA2 = 0.88 (Fig. 6b).

**Fig. 7:**
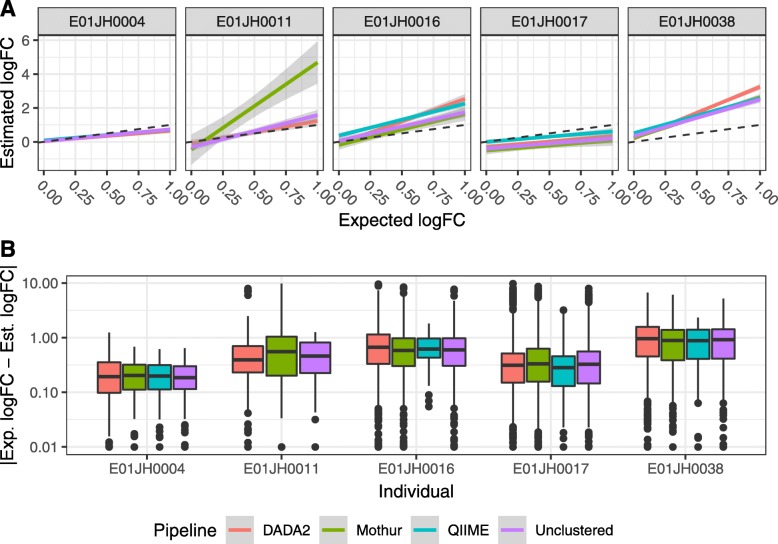
Differential abundance quantitative assessment. **a** Linear model of the relationship between estimated and expected log fold-change relative abundance between titrations for PRE-specific and PRE-dominant features by pipeline and individual, line color indicates pipelines. Dashed gray line indicates expected 1-to-1 relationship between the estimated and expected log fold-change. **b** Log fold-change error (|exp-est|) distribution by pipeline and individual. The QIMME E01JH0011 dataset did not contain any PRE-specific or PRE-dominant features required and was therefore excluded from this analysis

**Differential abundance assessment** The agreement between log-fold change estimates and expected values were individual-specific and consistent across pipelines (Fig. 7a). The individual-specific effect can be attributed to the fact that inferred *θ* values were not used to calculate expected values (unlike the relative abundance assessment.) Inferred *θ* values were not used to calculate the log-fold change expected values because all of the titrations and the *θ* estimates for the higher titrations were not monotonically decreasing. Using the inferred *θ* resulted in unrealistic expected log fold-change values, e.g., negative log-fold changes for PRE-specific features. The log-fold change estimates and expected values were consistent across pipelines with one notable exception: for subject E01JH0011, the Mothur log fold-change estimates were less consistent with expected values than the other pipelines. However, as *θ* was not corrected for differences in the proportion of prokaryotic DNA between the unmixed PRE and POST samples, we cannot say whether Mothur’s performance was worse than the other pipelines.

The log fold-change error distribution was consistent across pipelines (Fig. 7b). Additionally, we compared error distributions for log-fold change estimates using different normalization methods. Error rate distributions, including their long tails, were consistent across normalization methods. Seeing as the long tail was observed for the unclustered data as well, the log-fold change estimates contributing to the long tail are likely due to a bias associated with the molecular aspects of the measurement process and not the computational aspects.

**Fig. 8:**
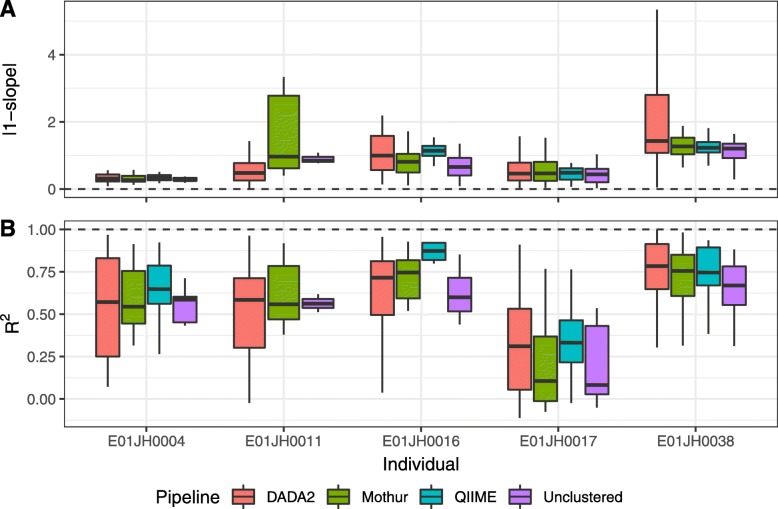
Feature-level differential abundance assessment. Log-fold change error bias (**a**) and variance (**b**) metric distribution by subject and pipeline. The bias (1−slope) and variance (*R*^2^) metrics are derived from the linear model fit to the estimated and expected log fold-change values for individual features. Boxplot outliers, 1.5×IQR from the median were excluded from the figure to prevent extreme metric values from obscuring metric value visual comparisons. As the QIMME E01JH0011 dataset did not contain any PRE-specific or PRE-dominant features is was not included in this analysis

Feature-level log fold-change bias and variance metrics were used to compare pipeline performance (Fig. 8). Feature-level bias and variance metrics are defined as the |1−slope| and *R*^2^ calculated from individual feature linear models of the estimated and expected log fold-change across all titration comparisons. For the bias metric, |1−slope|, the desired value is 0 (i.e., log fold-change estimate = log fold-change expected). The linear model *R*^2^ value was used to characterize the feature-level log fold-change variance as it indicates consistency between log fold-change estimates and expected values across titration comparisons. To compare bias and variance metrics across pipelines, mixed-effects models were used. The log fold-change bias metric was significantly different between pipelines (*F* = 4.62, *p*= 0.0103, 0.08, Fig. 8a), but the variance metric was not (*F* = 1.77, *p* = 0.17, Fig. 8b). QIIME had the lowest bias estimate (0.65) followed by Mothur (0.84) and DADA2 had the highest estimate (1.09). The QIIME and DADA2 estimates were significantly different from each other (*t* =−2.629, *p* = 0.022).

We should note that while edgeR uses a prior to moderate its logFC estimates, we did not use a prior to calculate the logFC expected values. To verify our conclusions are robust to this use of a prior, we also calculated the logFC estimates using multiple priors (Fig. S10 and S11). The prior had little effect on the differential abundance assessment results.

## Discussion

We developed a novel assessment framework utilizing a mixture dataset for evaluating marker-gene-survey computational methods (Fig. 1). Previous studies have used mixtures of environmental samples to assess RNAseq and microarray gene expression measurements [19–21], but this is the first study using mixtures to assess microbiome measurements.

When mixtures of environmental samples are used for assessment, expected values are calculated using information from unmixed samples and the mixture design. Our assessment dataset follows a two-sample titration mixture design, where DNA collected from five vaccine trial participants before and after exposure to pathogenic *Escherichia coli* was mixed following a log_2_ dilution series (Fig. 2). We assessed count table qualitative characteristics using count table sparsity and relative abundance information for features observed only in titrations (titration-specific) and unmixed samples (unmixed-specific) (Fig. 1b). We used statistical tests to determine if random sampling could account for the absence of unmixed-specific features from titrations or absence of titration-specific features from unmixed. We assessed count table quantitative characteristics using relative abundance and differential abundance error rate and feature-level bias and variance metrics (Fig. 1c).

**Count table assessment demonstration** We demonstrated our assessment framework on count tables generated by three commonly used bioinformatic pipelines, QIIME, Mothur, and DADA2 (Table 2). The objective of any pipeline is differentiating true biological sequences from artifacts introduced by the measurement process and arrive at accurate abundance estimates. Our qualitative assessment results, when combined with sparsity information provides a new method for evaluating how well bioinformatic pipelines account for sequencing artifacts without loss of true biological sequences.

The qualitative assessment evaluates if titration- and unmixed-specific features can be accounted for by random sampling alone (Fig. 1b). Titration- and unmixed-specific features not accounted for by sampling are measurement process artifacts. These artifacts are false-positives, not representative of actual sequences in a sample, or false-negatives, actual sequences in a sample not represented by count table features. Artifacts result from PCR errors such as chimeras, reads with high sequencing error rates, or cross sample contamination [25–27]. Count table sparsity information (the proportion of zero-valued cells) provides additional insight into the qualitative assessment results.

A high false-negative rate provides an explanation for DADA2’s high proportion of artifactual titration- and unmixed-specific features and count table having comparable sparsity to the other pipelines despite having significantly fewer features (Fig. S5 and Table 1). The DADA2 feature inference algorithm may be aggressively grouping lower abundance true sequences with higher abundance sequences. As a result, the low abundance sequences are not present in samples leading to increased sparsity and high abundance of unmixed- and titration-specific features. This aggressive grouping of sequences is a design choice made by the algorithm developers. The DADA2 documentation states that the default setting for OMEGA_A is conservative to prevent false-positives at the cost of increasing false-negatives [8]. Using the qualitative assessment methods described here, a user can adjust the OMEGA_A parameter to obtain a false-negative rate appropriate for their study.

Our assessment results (Table 2), suggest using DADA2 for feature-level abundance analysis, e.g., differential abundance testing. While DADA2 performed poorly in our qualitative assessment, the pipeline performed better in the quantitative assessment compared to the other pipelines. Additionally, the DADA2 poor qualitative assessment results due to false-negative features are unlikely to negatively impact feature-level abundance analysis. When determining which pipeline to use for a study, users should consider whether minimizing false-positives (by using DADA2) or false -negatives (by using Mothur) is more appropriate for their study objectives. Based on our findings we find that users of DADA2 can be more confident that an observed feature represents a member of the microbial community and not a measurement artifact, but careful examination of sequences assigned to features of interest should still be performed to ensure that low abundance features were not incorrectly clustered with high abundance features.

Limitations were observed for all the pipelines assessed in this study. To address these limitations the assessment framework presented here can be used for pipeline design optimization. For example the assessment framework can be used to evaluate the impact of using alternative paired-end alignment methods in the QIIME pipeline on the read drop-out rate.

**Using mixtures to assess 16S rRNA sequencing—lessons learned**

There are limitations in the use of our assessment dataset, these include (1) lack of agreement between the proportion of prokaryotic DNA from the unmixed samples in the titrations and the mixture design. (2) The mixture design resulted in a limited number of features and range of expected log-fold changes. These limitations are described below along with recommendations for addressing them in future studies.

Differences in the proportion of prokaryotic DNA in the samples used to generate the two-sample titrations series resulted in differences between the true mixture proportions and mixture design. We attempted to account for differences in mixture proportion from mixture design by using sequence data to estimate mixture proportions similar to how mRNA proportions in RNA samples were used in a previous mixture study [19]. We used an assay targeting the 16S rRNA gene to detect changes in the concentration of prokaryotic DNA across titrations, but were unable to quantify the proportion of prokaryotic DNA in the unmixed samples using qPCR data. Using the 16S rRNA sequencing data, we inferred the proportion of prokaryotic DNA from the POST sample in each titration. However, the uncertainty and accuracy of the inference method are not known, resulting in an unaccounted for source of error.

A better method for quantifying sample prokaryotic DNA proportion or using samples with consistent proportions would increase confidence in the expected value and, in-turn, error metric accuracy. Limitations in the prokaryotic DNA qPCR assay’s concentration precision limits the assay’s suitability for use in mixture studies. Digital PCR provides a more precise alternative to qPCR and is, therefore, a more appropriate method. Synthetic spike-ins developed to quantify microbial absolute abundance and correct for microbial load differences is another method that could be used to quantifying sample prokaryotic DNA proportions [28, 29]. Alternatively, using samples where the majority of the DNA is prokaryotic would minimize this issue. Mixtures of environmental samples can also be used to assess shotgun metagenomic methods as well. As shotgun metagenomics is not a targeted approach; differences in the proportion of prokaryotic DNA in a sample would not impact the assessment results in the same way as 16S rRNA marker-gene-surveys.

Using samples from a vaccine trial allowed for the use of a specific marker with an expected response, *E. coli*, during methods development. However, the high level of similarity between the PRE and POST unmixed samples resulted in a limited number of features for our the quantitative assessment. Using more diverse samples to generate mixtures would address this issue. Alternatively, instead of mixing PRE and POST samples from the same individual, mixing PRE and POST samples from different individuals would have resulted in additional features for use in our quantitative assessment. While unmixed sample similarity impacts the number of features that can be used in the quantitative assessment, the qualitative assessment is not impacted by unmixed sample similarity. Finally, a symmetric mixture design, for example, one with unmixed PRE and POST ratios of 1:4, 1:2, 1:1, 2:1, and 4:1, would provide a larger dynamic range of abundance values for assessing both PRE and POST-specific features.

## Conclusions

Our assessment framework can be used to evaluate and characterize 16S rRNA marker-gene survey analysis methods, in particular count tables produced by any 16S rRNA bioinformatic pipeline. We demonstrated our assessment framework with three commonly used bioinformatic pipelines. Our qualitative assessment results indicated that the QIIME and Mothur pipelines produced count tables with more false-positive features whereas the DADA2 count tables had more false-negative features. Overall, the three pipelines performed well in our quantitative assessment. Nevertheless, feature-level results for any 16S rRNA marker-gene survey should be interpreted with care. Improving quantitative and qualitative consistency requires advances in both the molecular biology and computational components of the measurement process.

## Methods

### Assessment framework

To assess the qualitative and quantitative performance of marker-gene survey analysis methods we developed a framework utilizing our two-sample titration dataset (Fig. 1). The qualitative assessment evaluates feature presence-absence. The quantitative assessment evaluates feature relative and differential abundance.

**Assessment dataset—mixture design** We developed a dataset with real-world complexity and expected values for method assessment using mixtures of environmental samples. The mixtures were generated from samples collected at multiple timepoints during an Enterotoxigenic *E. coli* (ETEC) vaccine trial [30] (Fig. 2). Samples from five trial participants were selected for our mixture dataset. We mixed samples collected prior to (PRE) and after (POST) ETEC exposure following a two-sample titration mixture design. We selected trial participants (individuals) and sampling time points based on *E. coli* abundance data collected using qPCR and 16S rRNA sequencing from Pop et al. [22]. For our dataset, we identified five individuals with no *E. coli* detected in samples collected from trial participants prior to ETEC exposure (PRE). Post ETEC exposure (POST) samples were identified as the timepoint after exposure to ETEC with the highest *E. coli* concentration for each subject (Fig. 2a). Due to limited sample availability, for E01JH0016, the timepoint with the second highest *E. coli* concentration was used as the POST sample. Independent titration series were generated for each subject. POST samples were titrated into PRE samples with POST proportions of 1/2, 1/4, 1/8, 1/16, 1/32, 1/1024, and 1/32,768 (Fig. 2b). Unmixed (PRE and POST) sample DNA concentration was measured using NanoDrop ND-1000 (Thermo Fisher Scientific Inc. Waltham, MA USA). Unmixed samples were diluted to 12.5 *ng*/*μL* in tris-EDTA buffer before mixing. The resulting titration series was composed of 45 samples, 7 titrations, and 2 unmixed samples for each of the 5 subjects.

The 45 samples were processed using the Illumina 16S library protocol (16S Metagenomic Sequencing Library Preparation, posted date November 27, 2013, downloaded from https://support.illumina.com). This protocol specifies an initial PCR of the 16S rRNA gene, followed by a sample indexing PCR, sample concentration normalization, and sequencing.

A total of 192 16S rRNA PCR assays were sequenced across 2 96-well plates including 4 PCR replicates per sample and 12 no-template controls. The initial PCR assay targeted the V3–V5 region of the 16S rRNA gene, Bakt_341F, and Bakt_806R [14]. The V3–V5 region is 464 base pairs (bp) long, with forward and reverse reads overlapping by 136 bp, using 2 × 300 bp paired-end sequencing [31] (http://probebase.csb.univie.ac.at). Primer sequences include overhang adapter sequences for library preparation (forward primer 5’- TCG TCG GCA GCG TCA GAT GTG TAT AAG AGA CAG CCT ACG GGN GGC WGC AG - 3’ and reverse primer 5’- GTC TCG TGG GCT CGG AGA TGT GTA TAA GAG ACA GGA CTA CHV GGG TAT CTA ATC C - 3’). The 16S rRNA gene was PCR amplified using the Kapa HiFi HotStart ReadyMix reagents (KAPA Biosystems, Inc. Wilmington, MA, USA) and product amplicon size was verified using agarose gel electrophoresis. Concentration measurements were made after the initial 16S rRNA PCR, the indexing PCR, and normalization steps. DNA concentration was measured using the QuantIT Picogreen dsDNA Kit (Cat # P7589, ThermoFisher Scientific) and fluorescent measurements were made with a Synergy2 Multi-Detection MicroPlate Reader (BioTek Instruments, Inc, Winooski, VT, USA).

Initial PCR products were purified using 0.8X AMPure XP beads (Beckman Coulter Genomics, Danvers, MA, USA) following the manufacturer’s protocol. After purification, the 192 samples were indexed using the Illumina Nextera XT index kits A and D (Illumina Inc., San Diego CA, USA) and then purified using 1.12X AMPure XP beads. Prior to pooling, the purified sample concentration was normalized using SequalPrep Normalization Plate Kit (Catalog n. A10510-01, Invitrogen Corp., Carlsbad, CA, USA), according to the manufacturer’s protocol. Pooled library concentration was checked using the Qubit dsDNA HS Assay Kit (Part# Q32851, Lot# 1735902, ThermoFisher, Waltham, MA, USA). Due to the low pooled amplicon library DNA concentration, a modified protocol for low concentration libraries was used. The library was run on an Illumina MiSeq, and base calls were made using Illumina Real Time Analysis Software version 1.18.54. The sequence data were deposited in the NCBI SRA archive under Bioproject PRJNA480312. Individual SRA run accession numbers and metadata in Supplemental Table. The sequencing data presented in this manuscript are part of a larger study and the SRX accession numbers should be used to access the specific data presented here. Sequencing data quality control metrics were computed using the Bioconductor Rqc package [32, 33].

Sequence data were processed using four bioinformatic pipelines: a de novo clustering method - Mothur [7], an open-reference clustering method - QIIME [5], and a sequence inference method—DADA2 [8], and unclustered sequences as a control. The code used to run the bioinformatic pipelines is available at https://github.com/nate-d-olson/mgtst_pipelines.

The Mothur pipeline follows the developer’s MiSeq SOP [7, 23]. The pipeline was run using Mothur version 1.37 (http://www.mothur.org/). We sequenced a larger 16S rRNA region, with smaller overlap between the forward and reverse reads, than the 16S rRNA region the SOP was designed for. Pipeline parameters were modified to account for differences in overlap and are noted for individual steps below. The Makefile and scripts used to run the Mothur pipeline are available https://github.com/nate-d-olson/mgtst_pipelines/blob/master/code/mothur. The Mothur pipeline included an initial preprocessing step where the forward and reverse reads are trimmed and filtered using base quality scores and were merged into single contigs for each read pair. The following parameters were used for the initial contig filtering, no ambiguous bases, max contig length of 500 bp, and max homopolymer length of 8 bases. For the initial read filtering and merging step, low-quality reads were identified and filtered from the dataset based on the presence of ambiguous bases, failure to align to the SILVA reference database (V119, https://www.arb-silva.de/) [34], and identification as chimeras. Prior to alignment, the SILVA reference multiple sequence alignment was trimmed to the V3–V5 region, positions 6388 and 25,316. Chimera filtering was performed using UChime (version v4.2.40) without a reference database [25]. OTU clustering was performed using the OptiClust algorithm with a clustering threshold of 0.97 [6]. The RDP classifier implemented in Mothur was used for taxonomic classification against the Mothur provided version of the RDP v9 training set [35].

The QIIME open-reference clustering pipeline for paired-end Illumina data was performed according to the online tutorial (Illumina Overview Tutorial (an IPython Notebook): open reference OTU picking and core diversity analyses, http://qiime.org/tutorials/) using QIIME version 1.9.1 [5]. Briefly, the paired-end reads were merged using fastq-join (version 1.3.1, [36]) and open-reference clustering was performed using the Usearch algorithm [37] with Greengenes database version 13.8 with a 97% similarity threshold [38]. The bash script used to run the QIIME pipeline is available at https://github.com/nate-d-olson/mgtst_pipelines/blob/master/code/qiime_pipeline.sh.

DADA2, an R native pipeline, was also used to process the sequencing data [8]. The forward and reverse reads were independently quality filtered and grouped using the DADA2 probability model. Independently grouped forward and reverse reads were then merged and chimeras were filtered. Taxonomic classification was performed using the DADA2 implementation of the RDP naïve Bayesian classifier [35] and the SILVA database V123 provided by the DADA2 developers [34,https://benjjneb.github.io/dada2/training.html]. Code for running the DADA2 pipeline is available at https://github.com/nate-d-olson/mgtst_pipelines/blob/master/code/dada2_pipeline.R.

The unclustered pipeline was based on the Mothur de novo clustering pipeline, where the paired-end reads were merged, filtered, and dereplicated. Reads were aligned to the reference Silva alignment (V119, https://www.arb-silva.de/), and reads failing alignment were excluded from the dataset. Taxonomic classification implemented in Mothur was performed using the Mothur implemented RDP classifier implemented and the RDP v9 training set provided by the Mothur developers. To limit the dataset size, only the most abundant 40,000 OTUs (comparable to the Mothur dataset) across all samples were used.

**Qualitative assessment** The qualitative assessment evaluates feature presence absence by count table sparsity and artifactual feature proportion. Count table sparsity is the proportion of 0 valued cells in the count table. Artifactual feature proportion is the proportion of unmixed- and titration-specific features with observed abundance values not explained by sampling alone. Unmixed-specific features are features only observed in the unmixed PRE or POST samples (Fig. 1b). Titration-specific features are features only observed in the titrations. Unmixed- and titration-specific features can arise from errors in PCR/sequencing, feature inference processes, or differences in sampling depth. The artifactual feature proportion provides context for interpreting count table sparsity results, where low artifactual feature proportion and high sparsity is indicative of a high false-positive rate and high artifactual feature proportion, and high sparsity indicates a high false-negative rate (Fig. 1c).

Hypothesis tests were used to determine if random sampling alone, here sequencing depth, could account for unmixed- and titration-specific features. *p* values were adjusted for multiple comparisons using the Benjamini and Hochberg method [39]. For unmixed-specific features, a binomial test was used to evaluate if true feature relative abundance is less than the expected relative abundance. The binomial test was infeasible for titration-specific features. Count table abundance values for the titration-specific features was 0 in the unmixed samples, their estimated probability of occurrence, *π*_min_, is equal to 0, and thus, the binomial test fails. Therefore, we formulated a Bayesian hypothesis test for titration-specific features defined in Eq. 2. This Bayesian approach evaluated if the true feature proportion is less than the minimum detected proportion. When assuming equal priors, *P*(*π*<*π*_min_)=*P*(*π*>*π*_min_), (2) reduces to (3). We define *π* as the true feature proportion, *π*_min_ the minimum detected proportion, *C* the expected feature counts, and *C*_obs_ the observed feature counts. Count values for *C* were simulated using a beta prior (with varying alpha and beta values) for *π*>*π*_min_ and a uniform distribution for *π*<*π*_min_. Higher values of alpha and beta will skew the prior right and left, respectively. Our Bayesian hypothesis tests (3) results were largely unaffected by beta distribution parameterization (Fig. S4). *π*_min_ was calculated using the mixture Eq. 1 where *q*_pre,*j*_ and *q*_post,*j*_ are min(**Q**_pre_) and min(**Q**_post_) across all features for a subject and pipeline. Our assumption is that *π* is less than *π*_min_ for features not observed in unmixed samples. Artifacts not explained by sequencing alone are likely false-positives or false-negatives due to errors in the sequence measurement and inference processes.

2 $$ \begin{aligned} p & = P(\pi < \pi_{{\text{min}}} | C \geq C_{{\text{obs}}}) \\ & = \frac{P(C \geq C_{{\text{obs}}}| \pi < \pi_{{\text{min}}})P(\pi < \pi_{{\text{min}}})}{P(C\! \geq\! C_{{\text{obs}}}| \pi \!<\! \pi_{{\text{min}}})P(\pi \!< \!\pi_{{\text{min}}})\! \,+\, P(C\! \geq \!C_{{\text{obs}}}| \pi\! \geq\! \geq\! \pi_{{\text{min}}})P(\!\pi\! \geq\! \pi_{{\text{min}}})} \\ \end{aligned}  $$

3 $$ p = \frac{P(C \geq C_{{\text{obs}}}| \pi < \pi_{{\text{min}}})}{P(C \geq C_{{\text{obs}}})}  $$

**Quantitative assessment** For the quantitative assessment, we compared the observed relative abundance and log fold-changes to expected values derived from the titration experimental design. For the observed values in our relative abundance assessment, we averaged feature relative abundance across PCR replicates. Feature average relative abundance across PCR replicates was calculated using a negative binomial model. We used the averaged relative abundance across PCR replicates as the observed relative abundance values *obs* for the relative abundance assessment. Average relative abundance values were used to prevent PCR replicate outliers from biasing the assessment results. Equation (1) and inferred *θ* values were used to calculate the expected relative abundance values (*exp*). We defined the relative abundance error rate as |exp−obs|/exp. We developed bias and variance metrics to assess feature performance. The feature-level bias and variance metrics were defined as the median error rate and robust coefficient of variation (RCOV=IQR/median), respectively.

We assessed differential abundance estimates by comparing log fold-change between samples in the titration series including PRE and POST to the expected log fold-change values. Log fold-change estimates were calculated using EdgeR [40, 41]. Expected log fold-change values for feature *j* between titrations *l* and *m* was calculated using Eq. (4), where *θ* was the proportion of POST bacterial DNA in a titration, and *q* is feature relative abundance. For features only present in PRE samples, *PRE-specific*, the expected log fold-change was independent of the observed counts for the unmixed samples and was calculated using Eq. (5). For features only present in POST samples, *POST-specific*, the expected log fold-change values can be calculated in a similar manner. However, POST-specific features were rarely observed in more than one titration and therefore not included in this analysis.

Due to a limited number of PRE-specific features, both PRE-specific and PRE-dominant features were used in the differential abundance assessment. PRE-specific features were defined as features observed in all four PRE PCR replicates and not observed in any of the POST PCR replicates and PRE-dominant features were also observed in all four PRE PCR replicates and observed in one or more of the POST PCR replicates but with a log fold-change greater than 5 between PRE and POST samples.

4 $$ {\text{logFC}}_{lm,j} = \log_{2}\left(\frac{\theta_{l} q_{{\text{post}},j} + (1 - \theta_{l}) q_{{\text{pre}},j}}{\theta_{m} q_{{\text{post}},j} + (1 - \theta_{m}) q_{{\text{pre}},j}}\right)  $$

5 $$ {\text{log}}FC_{lm,j} = {\text{log}}_{2}\left(\frac{1-\theta_{l}}{1-\theta_{m}}\right)  $$

### Count table assessment demonstration

We demonstrated the assessment framework by comparing the qualitative and quantitative assessment results across the three pipelines. We first characterized overall differences in the count tables produced by the pipelines. This characterization included total number of features, total abundance by individual, dropout-rate, and taxonomic composition.

**Qualitative assessment** For the qualitative assessment, we compared the proportion of artifactual features. The artifactual feature proportion was defined as the proportion of unmixed- and titration-specific features with abundance values that could not be explained by sampling alone. These are PCR replicates with *p* values less than 0.05 after multiple hypothesis test correction for the binomial and Bayesian hypothesis tests described in the “Assessment framework” section. We additionally used the count table sparsity values to identify potential mechanisms responsible for differences in artifactual feature proportions.

**Quantitative assessment** Mixed-effects models with individual as a random effect were used to compare feature-level error rate bias and variance metrics across pipelines. Extreme feature-level error rate bias and variance metric outliers were excluded from this analysis to minimize biases due to poor model fit. Features with large bias and variance metrics, 1.5×IQR from the median, were deemed outliers.

We fit the following mixed effect model to test for differences in measurement bias across pipelines

$$e_{ijk} = b + b_{i} + z_{j} + \epsilon_{ijk} $$

where *e*_*ijk*_ was the observed error across features and titrations *k* for pipeline *i* on individual *j*. *b*_*i*_ was a fixed term modeling the pipeline effect, *z*_*j*_ was a random effect (normally distributed with mean 0) capturing overall bias differences across individuals. We fit a similar model for differences in error variance across pipelines.

We used estimated terms $\hat {b}_{i}$ from the mixed effects model to test for pair-wise differences between pipelines. These multiple comparisons were performed with Tukey’s HSD test. A one-sided alternative hypothesis was used to determine which pipelines had smaller feature-level error rates.

## Supplementary information

**Additional file 1** Supplementary material.

**Additional file 2** Sample ID Error and Correction.

**Additional file 3** Supplemental Table.

## Data Availability

Sequence data was deposited in the NCBI SRA archive under Bioproject PRJNA480312. The sequence data presented in this manuscript are part of a larger study where the same set of sample were sequenced in quadruplicate. Individual SRA run accession numbers and metadata are in Supplemental Table. Use the SRX acccession numbers in the supplemental table to retrieve the sequence data presented in this manuscript. The code used to run the bioinformatic pipelines is available at https://github.com/nate-d-olson/mgtst_pipelines. Scripts used to analyze the data are available at https://github.com/nate-d-olson/abundance_assessment.
