# Supplementary material for: A framework for assessing 16S rRNA marker-gene survey data analysis methods using mixtures
Source: Microbiome. 2020 Mar 13;8:35. doi: 10.1186/s40168-020-00812-1 (PMC7071580; doi:10.1186/s40168-020-00812-1)
Supplement: Supplementary file 1 — Additional file 1 Supplementary material. [file 40168_2020_812_MOESM1_ESM.pdf]

Supplemental Material - A framework for assessing 16S marker gene survey data analysis methods using mixtures.

Olson et al

October 5, 2019

Titration Series Validation

Methods

To increase confidence in the expected values used in our assessment framework we validated the proportion of unmixed samples measured by the 16S rRNA marker-gene sequencing assay. To correct for observed deviation from our mixture design we estimated the proportion of unmixed POST in the titrations using the 16S rRNA sequencing data.

Volumetric Mixing Validation qPCR was used to validate volumetric mixing and check for differences in the proportion of prokaryotic DNA across titrations (Fig. 1). To ensure the two-sample titrations were volumetrically mixed according to the mixture design, independent ERCC plasmids were spiked into the unmixed PRE and POST samples [1] (NIST SRM SRM 2374) (Table 1). The ERCC plasmids were resuspended in 100 μL tris-EDTA buffer and 2 μL of resuspended plasmids was spiked into the appropriate unmixed sample, with a final concentration of approximately $2.5X10^{10} copies/\mu L$. Plasmids were spiked into 100 μL unmixed PRE and POST samples with normalized DNA concentration of 12.5 $ng/\mu L$. POST sample ERCC plasmid abundance was quantified using TaqMan gene expression assays (FAM-MGB, Catalog # 4448892, ThermoFisher) specific to each ERCC plasmid and TaqMan Universal MasterMix II (Catalog # 4440040,

ThermoFisher Waltham, MA USA). qPCR assays were perfomed in triplicate using the QuantStudio Real-Time qPCR (ThermoFisher). ERCCs were also spiked into PRE samples but were not used to validate volumetric mixing as PRE sample proportion differences were too small for qPCR quantification. The expected difference for the entire range of PRE concentrations is 1 C_t.

To check for differences in the proportion of bacterial DNA in the PRE and POST samples, bacterial DNA concentration in the titrations was quantified using the Femto Bacterial DNA quantification kit (Zymo Research, Irvine CA). All samples were run in triplicate along with an in-house *E. coli* DNA log_{10} dilution standard curve. Three concentrations were used for the in-house standard, 20 ng/ul, 2ng/ul, and 0.2 ng/ul, with 91.49 efficiency and 0.999 R^2. qPCR assays were performed using the QuantStudio Real-Time qPCR (ThermoFisher). Amplification data and Ct values were exported as tsv files using QuantStudio Design and Analysis Software v1.4.1. Statistical analysis was performed on the exported data using custom scripts in R [2]. The qPCR data and scripts used to analyze the data are available at `https://github.com/nate-d-olson/abundance_assessment`.

The following linear model (1) was used to infer the proportion of prokaryotic DNA, θ, in each titration. Where $\mathbf{Q}_i$ is a vector of titration i feature relative abundance estimates and $\mathbf{Q}_{pre}$ and $\mathbf{Q}_{post}$ are vectors of feature relative abundance estimates for the unmixed PRE and POST samples. Feature relative abundance estimates were calculated using a negative binomial model.

$$\mathbf{Q}_i = \theta_i(\mathbf{Q}_{post} - \mathbf{Q}_{pre}) + \mathbf{Q}_{pre} \tag{1}$$

To fit the model and prevent uninformative and low abundance features from biasing θ estimates, only features meeting the following criteria were used. To improve feature level model fit, features had to be observed in at least 14 of the 28 total titration PCR replicates (4 replicates per 7 titrations.) To increase confidence in PRE and POST abundance estimates the features were present in either all four or none of the PRE and POST PCR replicates. Finally, to eliminate uninformative features with no change in abundance across titrations only features with greater than 2-fold difference in relative abundance between the PRE and POST samples were used.

16S rRNA sequencing count data is known to have a non-normal mean-variance relationship resulting in poor model fit for standard linear regression [3]. Generalized linear models provide an alternative to standard least-

squares regression. The above model is additive and therefore θ_i cannot be directly inferred in log-space. To address this limitation, we fit a model to (1) using standard least-squares regression and obtained non-parametric 95 % confidence intervals for the θ estimates by bootstrapping with 1000 replicates. Bootstrapping was performed by resampling informative features, defined above, by subject.

Results

Volumetric Mixing Validation Titration series volumetric mixing was validated using qPCR to quantify exogenous DNA (ERCC plasmids) spiked into the POST samples prior to mixing. The expectation is that the ERCC plasmid copy number will change at a rate consistent with the change in proportion of POST along the titration series (Main Fig. 1B and 1). For our log_2 two-sample-titration mixture design the expected slope of the regression line between titration factor and Ct is 1, corresponding to a doubling in template DNA every PCR cycle. The qPCR assay standard curves had a high level of precision with R^2 values close to 1 and amplification efficiencies between 0.84 and 0.9 for all standard curves indicating the assays were suitable for validating the titration series volumetric mixing (Table 1). The qPCR assays targeting the ERCCs spiked into the POST samples had R^2 values and slope estimates close to 1 (Table 1). Slope estimates less than one were attributed to assay standard curve efficiency less than 1 (Table 1). When considering the quantitative limitations of the qPCR assay these results confirm that the unmixed samples were volumetrically mixed according to the two-sample titration mixture design.

Prokaryotic DNA Proportion Validation Observed changes in prokaryotic DNA concentration across titrations indicate the proportion of prokaryotic DNA from the unmixed PRE and POST samples in a titration is inconsistent with the mixture design (Fig. 2). A qPCR assay targeting the 16S rRNA gene was used to quantify the concentration of prokaryotic DNA in the titrations. If the proportion of prokaryotic DNA is the same between PRE and POST samples the slope of the concentration estimates across the two-sample titration would be 0. For subjects where the proportion of prokaryotic DNA is higher in the PRE samples, the slope will be negative, and positive when the proportion is higher for POST samples. The slope estimates are significantly different from 0 for all subjects excluding E01JH0011 (Fig. 2).

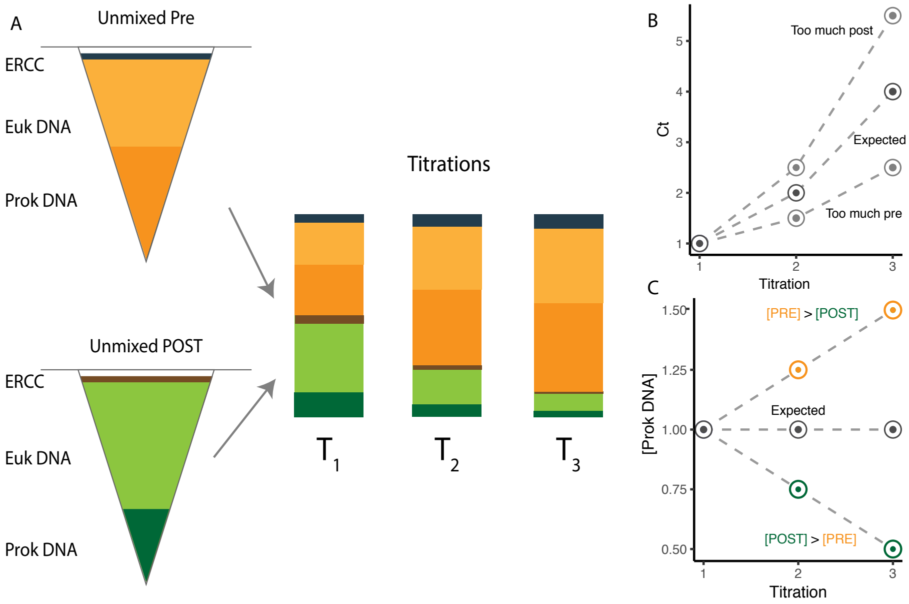

Figure 1: Titration-series validation methods. (A) ERCCs were spiked into unmixed PRE and POST DNA. Unmixed DNA is composed of prokaryotic and eukaryotic DNA. Unmixed DNA used to generate two-sample titration series. The proportion of prokaryotic DNA differs between PRE and POST resulting in the proportion of measureable PRE and POST DNA (prokarytic DNA) in the titrations differing from the mixture design. (B) Results from qPCR quantification of the ERCCs was used to validate volumetric mixing. (C) 16S rRNA qPCR is used to validate the prokaryotic DNA proportion. For B and C expectation is indicated in grey. Deviations from expectation along with explanations are indicated in light grey for B and orange and green for C.

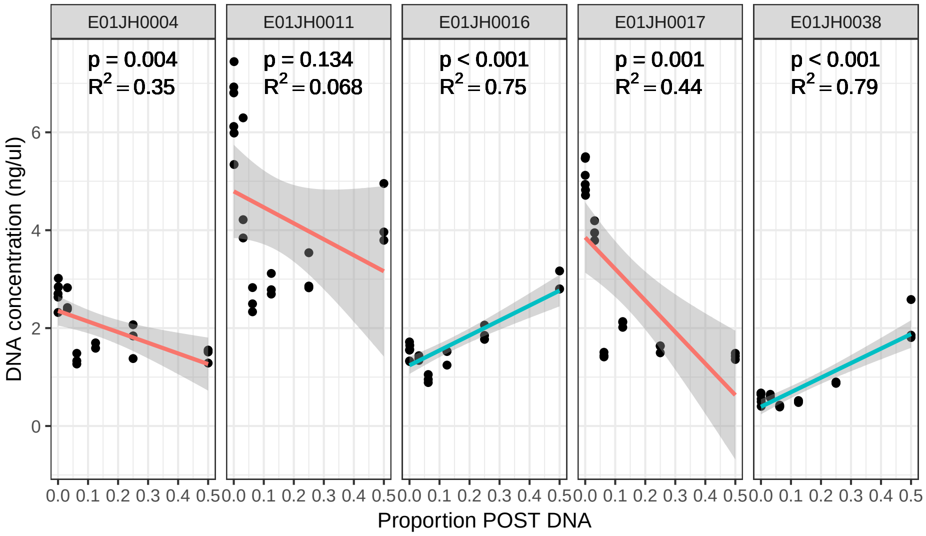

Figure 2: Prokaryotic DNA concentration (ng/ul) across titrations measured using a 16S rRNA qPCR assay. R^2 and p-values are linear models fit to prokaryotic DNA concentration versus proportion post DNA for each individual. Red and blue lines indicate negative and positive slope estimates respectively. p-value indicates significant difference from the expected slope of 0. The grey regions indicate the linear model 95% confidence interval. Multiple test correction was performed using the Benjamini-Hochberg method. One of the E01JH0004 PCR replicates for titration 3 ($\theta = 0.125$) was identified as an outlier, with a prokaryotic DNA concentration of 0.003 ng/ul, and was excluded from the linear model. The linear model slope was still significantly different from 0 when the outlier was included.

Table 1: ERCC Spike-in qPCR assay information and summary statistics. ERCC is the ERCC identifier for the ERCC spike-in, Assay is TaqMan assay, and Length and GC are the size and GC content of the qPCR amplicon. The Std. R^2 and Efficiency (E) statistics were computed for the standard curves. R^2 and slope for titration qPCR results for the titration series.

Subject	ERCC	Assay	Length	Std. R^2	E	R^2	Slope
E01JH0004	012	Ac03459877-a1	77	0.9996	86.19	0.98	0.92
E01JH0011	157	Ac03459958-a1	71	0.9995	87.46	0.95	0.90
E01JH0016	108	Ac03460028-a1	74	0.9991	87.33	0.95	0.84
E01JH0017	002	Ac03459872-a1	69	0.9968	85.80	0.89	0.93
E01JH0038	035	Ac03459892-a1	65	0.9984	86.69	0.95	0.94

These results indicate that the proportion of prokaryotic DNA is lower in POST when compared to the PRE samples for E01JH0004 and E01JH0017 and higher for E01JH0016 and E01JH0038.

Correcting for Deviations from Mixture Design Our titration validation results identified differences in the proportion of prokaryotic DNA in PRE and POST samples (Fig. 2). Therefore our expected values used in measurement assessment need to account for differences in the proportion of prokaryotic DNA from unmixed samples. To account for differences in prokaryotic DNA proportion we inferred the proportion of POST sample prokaryotic DNA in a titration, θ, using the 16S rRNA sequencing data (Fig. 3).

The relationship between the inferred and mixture design θ values were consistent across pipelines but not subject. For individual E01JH0004 the inferred and mixture design θ values were in agreement. For individual E01JH0017 the inferred values were consistently less than the mixture design values. For individuals E01JH0011, E01JH0016, and E01JH0038 the inferred values were consistently greater than the mixture design values.

The qPCR prokaryotic DNA results support the theta estimates. Differences between the estimated theta and mixture design theta are due to difference in the amount of prokaryotic DNA in the unmixed samples. E01JH0017 was the only individual with a higher concentration of prokaryotic DNA in the unmixed POST sample than the unmixed PRE sample. E01JH0017 was

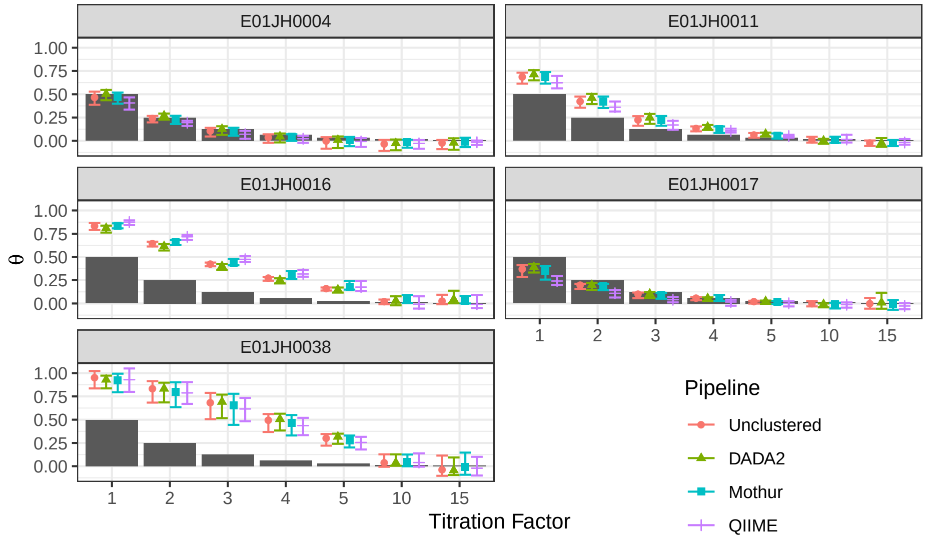

Figure 3: Theta estimates ($\hat{\theta}$) by titration, biological replicate, and bioinformatic pipeline. Points indicate mean of 1000 bootstrap θ estimates and error bars 95% confidence interval. Grey bars indicate expected θ values based on mixture design. Points above grey bars indicate the titrations with a high proportion of prokaryotic DNA from the POST sample than expected. Points below grey bars indicate titrations with high proportion of prokaryotic DNA from the PRE sample.

also the only with theta estimates lower than the mixture design theta value. The ratio of prokaryotic DNA between the PRE and POST samples corresponded with how well the estimated theta values agreed with the expected theta values. E01JH004 had the smallest PRE/POST prokaryotic DNA ration, 2.2, and E01JH0038 had the largest ratio, 23.0. (Fig. 2).

Qualitative Assessment of *Titration-Specific* Features using Beta Distribution Prior

To test whether our choice of uniform prior was misrepresenting the distribution of feature abundance simulated from, we ran the Bayesian hypothesis test given in equations (3) and (4) using a beta prior distribution under various parameterizations of shape parameters alpha and beta (Fig. 4). By weighting the beta parameter higher, the sampling distribution simulated will skew left, with a higher chance of simulating a low or null count, as is customary in microbiome data. If the alpha parameter is weighted higher, then the sampling distribution is skewed right, towards higher abundant features and a long tail of lower abundant features. We found that no matter the choice of beta prior, the results of the hypothesis tests were consistent: DADA2 finds far more titration specific features that cannot be explained by sampling error alone and are likely due to the conservative nature of this pipeline when it comes to binning reads into different features.

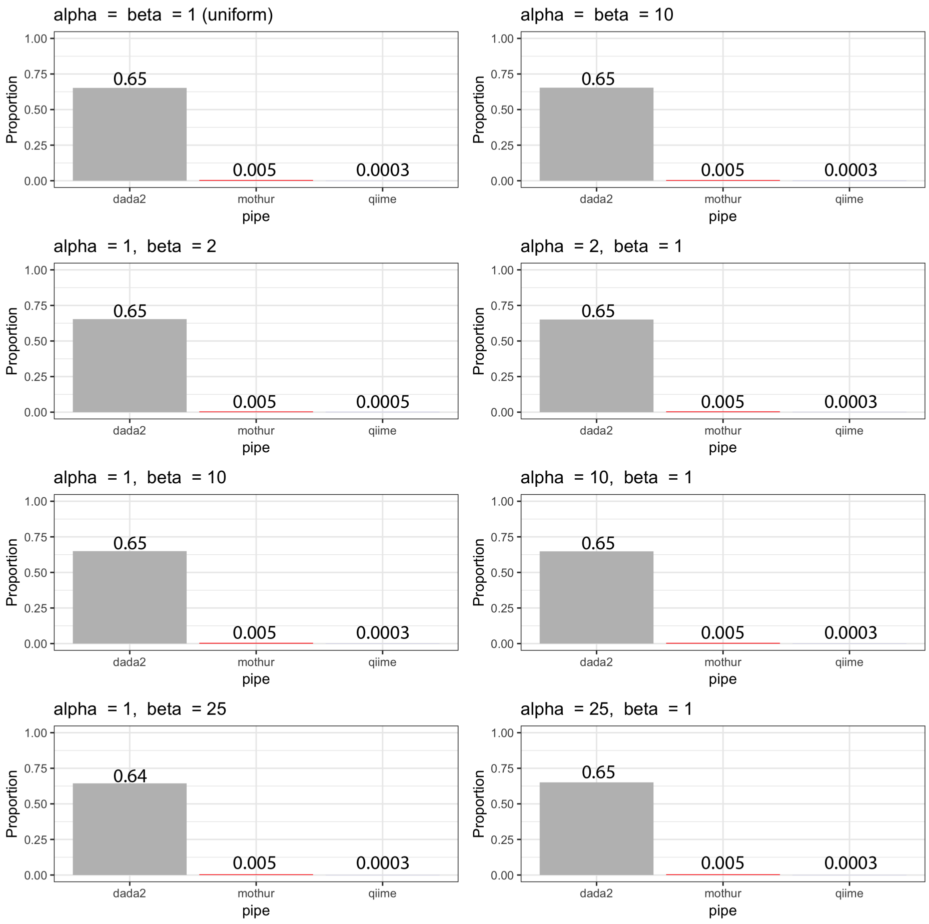

Figure 4: *Titration-specific* artifactual feature proportion. Artifactual features are those which could not be explained by sampling alone by pipeline and beta prior. Only artifactual feature proportions for subject E01JH0004 are shown. Plot titles indicate beta distribution parameters used in the bayesian hypothesis test detailed in equations (3) & (4) in the main text. Artifactual feature proportion values shown above each bar.

References

[1] Baker, S.C., Bauer, S.R., Beyer, R.P., Brenton, J.D., Bromley, B., Burrill, J., Causton, H., Conley, M.P., Elespuru, R., Fero, M., *et al.*: The external rna controls consortium: a progress report. Nature methods **2**(10), 731–734 (2005)

[2] R Core Team: R: A Language and Environment for Statistical Computing. R Foundation for Statistical Computing, Vienna, Austria (2018). R Foundation for Statistical Computing. https://www.R-project.org/

[3] McMurdie, P.J., Holmes, S.: Waste not, want not: why rarefying microbiome data is inadmissible. PLoS Comput. Biol. **10**(4), 1003531 (2014)

Supplemental Tables and Figures

Table 2: Sparsity levels for the three pipelines examined by individual (PRE and POST) and overall.

Individual	DADA2	Mothur	QIIME	Unclustered
E01JH0004	0.78	0.94	0.84	0.79
E01JH0011	0.76	0.94	0.84	0.80
E01JH0016	0.72	0.93	0.85	0.82
E01JH0017	0.74	0.94	0.86	0.82
E01JH0038	0.76	0.93	0.83	0.81
Overall	0.93	0.98	0.94	0.89

Table 3: Number of *titration-specific* and *unmixed-specific* features by pipeline and individual. Features is the total number of features. # Specific is the number of *titration-* or *unmixed-specific* features. # Artifactual is the number of specific features that were artifactual, $p < 0.05$, and Proportion is the # Artifactual / # Specific.

Pipeline	Individual	Features	Specificity Type	# Specific	# Artifactual	Proportion
DADA2	E01JH0004	922	titration	480	40	0.0833
			unmixed	120	19	0.1583
	E01JH0011	722	titration	376	30	0.0798
			unmixed	109	39	0.3578
	E01JH0016	835	titration	344	42	0.1221
			unmixed	123	20	0.1626
	E01JH0017	969	titration	485	48	0.0990
			unmixed	128	31	0.2422
	E01JH0038	901	titration	453	47	0.1038
			unmixed	107	22	0.2056
Mothur	E01JH0004	9954	titration	7172	3	0.0004
			unmixed	1788	0	0.0000
	E01JH0011	8473	titration	5878	4	0.0007
			unmixed	1715	7	0.0041
	E01JH0016	8639	titration	5966	2	0.0003
			unmixed	1744	1	0.0006
	E01JH0017	10504	titration	7891	4	0.0005
			unmixed	1647	1	0.0006
	E01JH0038	8237	titration	5664	6	0.0011
			unmixed	1587	1	0.0006
QIIME	E01JH0004	5553	titration	2684	0	0.0000
			unmixed	527	10	0.0190
	E01JH0011	4190	titration	1822	0	0.0000
			unmixed	584	26	0.0445
	E01JH0016	4377	titration	2096	1	0.0005
			unmixed	521	29	0.0557
	E01JH0017	5808	titration	3174	0	0.0000
			unmixed	521	20	0.0384
	E01JH0038	3920	titration	1835	0	0.0000
			unmixed	423	18	0.0426

Table 4: Maximum feature-level relative error rate bias (median error rate) and variance (robust COV) by pipeline and individual.

Metric	Pipeline	E01JH0004	E01JH0011	E01JH0016	E01JH0017	E01JH0038
	DADA2	2.06	1.14	0.75	2.25	0.66
Bias	Mothur	0.69	0.92	1.53	1.13	1.93
	QIIME	0.78	1.22	2.16	1.01	1.11
	DADA2	3.26	6.71	18.82	5.32	7.56
Variance	Mothur	3.11	5.27	4.27	4.02	6.07
	QIIME	4.89	5.82	9.02	8.71	7.55

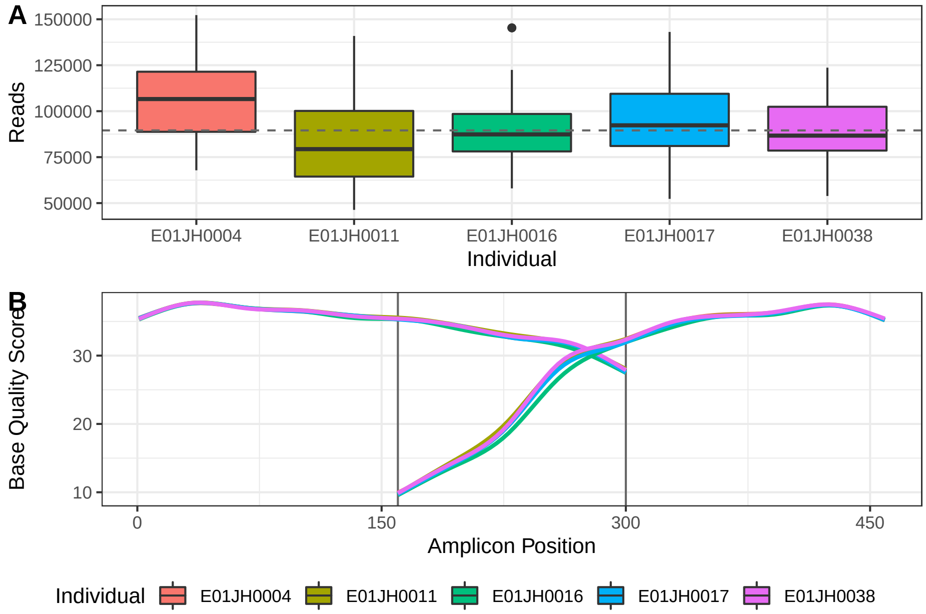

Figure 5: Sequence data set characteristics. (A) Distribution in the number of raw reads generated per barcoded sample (Library Size) by individual. Boxplots summarize data distribution with horizontal bar as median, boxes indicating interquartile range, whiskers $\pm 1.5 \times IQR$, and black points outliers. The dashed horizontal line indicates overall median library size. Excluding one PCR replicate from subject E01JH0016 titration 5 that had only 3,195 reads. (B) Smoothing spline of the base quality score (BQS) across the amplicon by subject. Vertical lines indicate approximate overlap region between forward and reverse reads. Forward reads go from position 0 to 300 and reverse reads from 464 to 164.

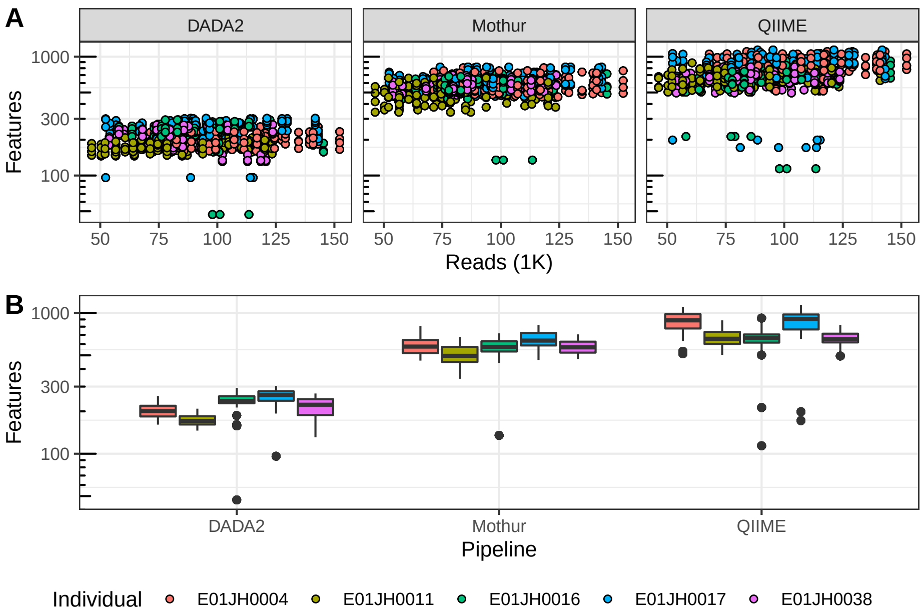

Figure 6: Relationship between the number of reads and features per sample by bioinformatic pipeline. (A) Scatter plot of observed features versus raw reads per sample. (B) Observed feature distribution by pipeline and individual. Excluding one PCR replicate from subject E01JH0016 titration 5 with only 3,195 reads, and the Mothur E01JH0017 titration 4 (all four PCR replicates), with 1,777 observed features.

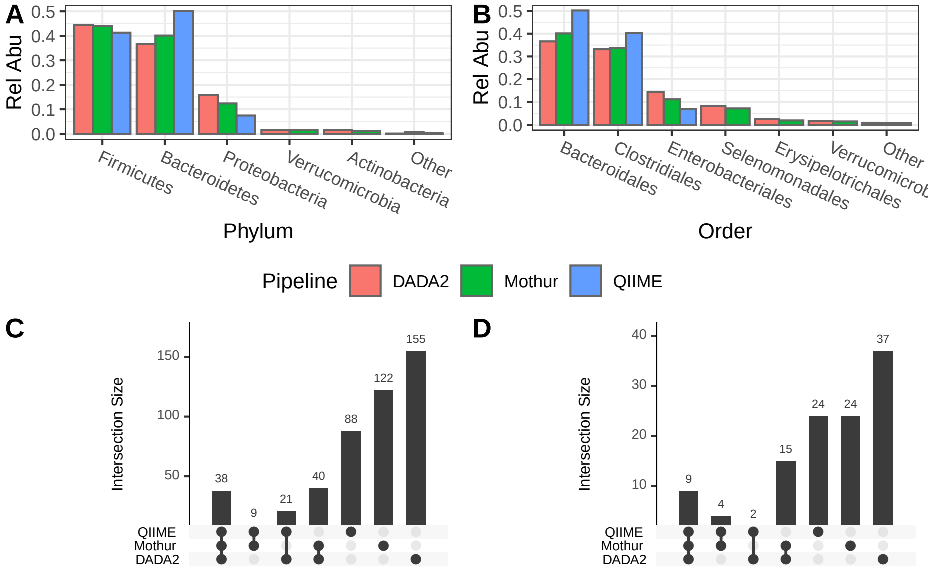

Figure 7: Comparison of dataset taxonomic composition across pipelines. Phylum (A) and Order (B) relative abundance by pipeline. Taxonomic groups with less than 1% total relative abundance were grouped together and indicated as other. Pipeline genus-level taxonomic assignment set overlap for all genera (C) and the upper quartile genera by relative abundance for each pipeline (D). Intersection size is the number of features observed in the pipeline combination indicated on the x-axis. For example in C, 39 genera are observed in all three pipelines and 88 are observed in only QIIME and not Mothur or DADA2.

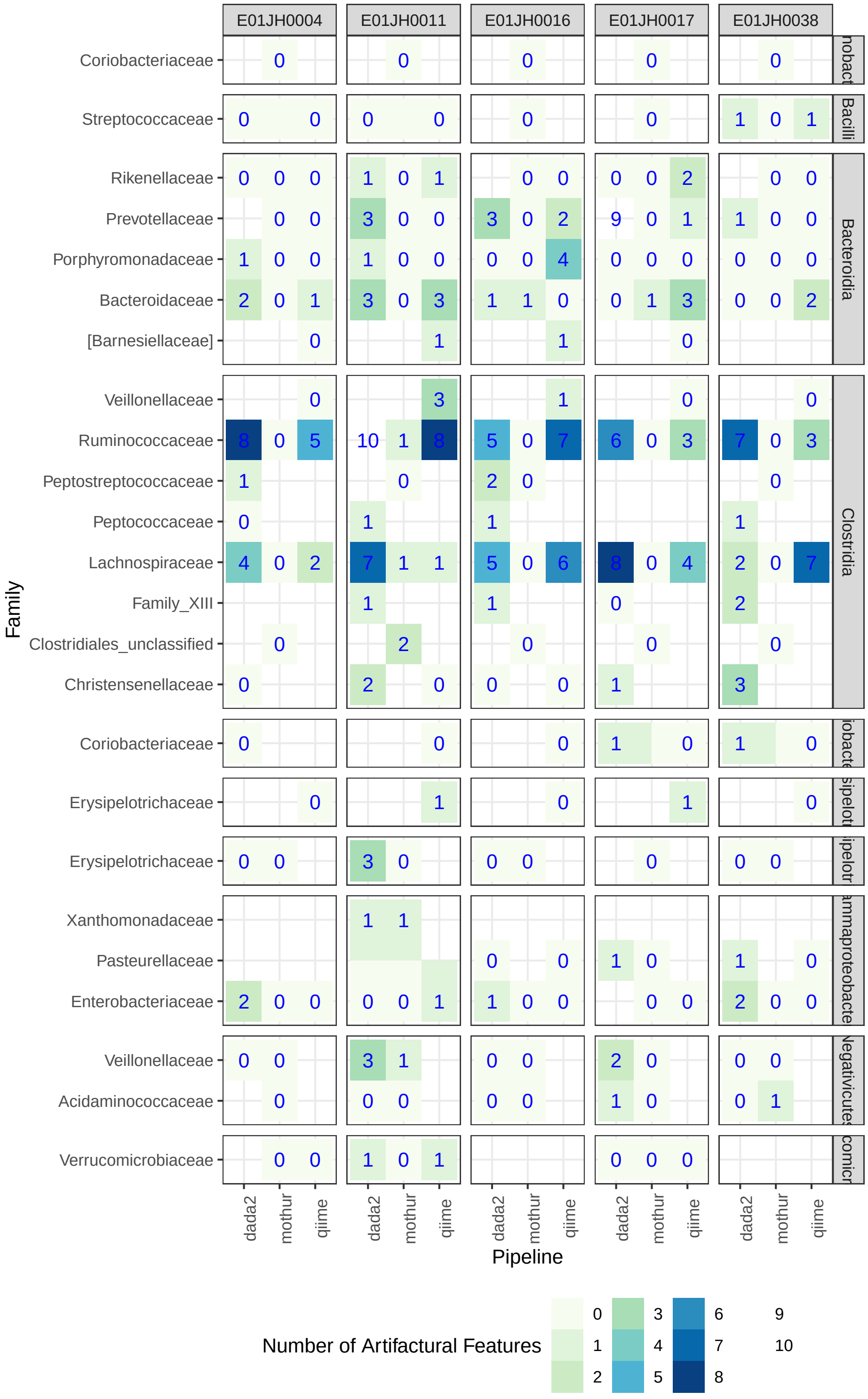

Figure 8: Frequency of titration-specific artifactual features by taxonomic family across bioinformatic pipeline and individual.

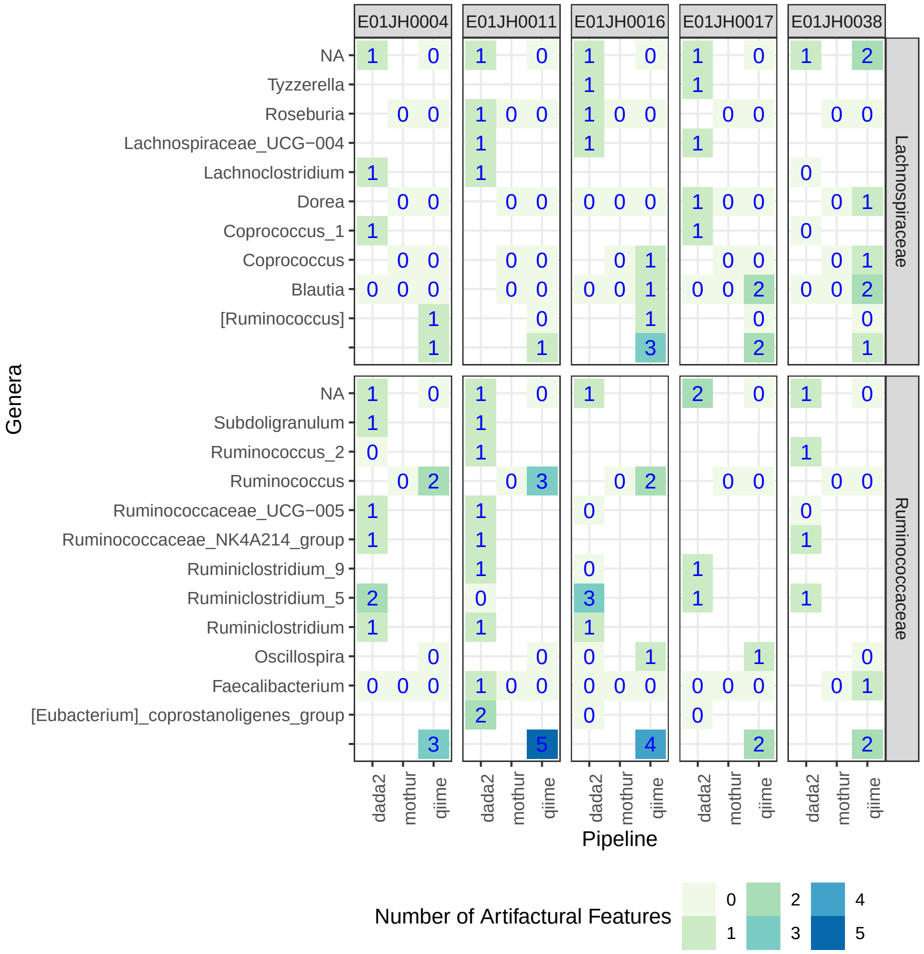

Figure 9: Frequency of titration-specific artifactual features by Genera across bioinformatic pipeline and individual for the two families with titration-specific artifactual features observed across both pipeline and individual.

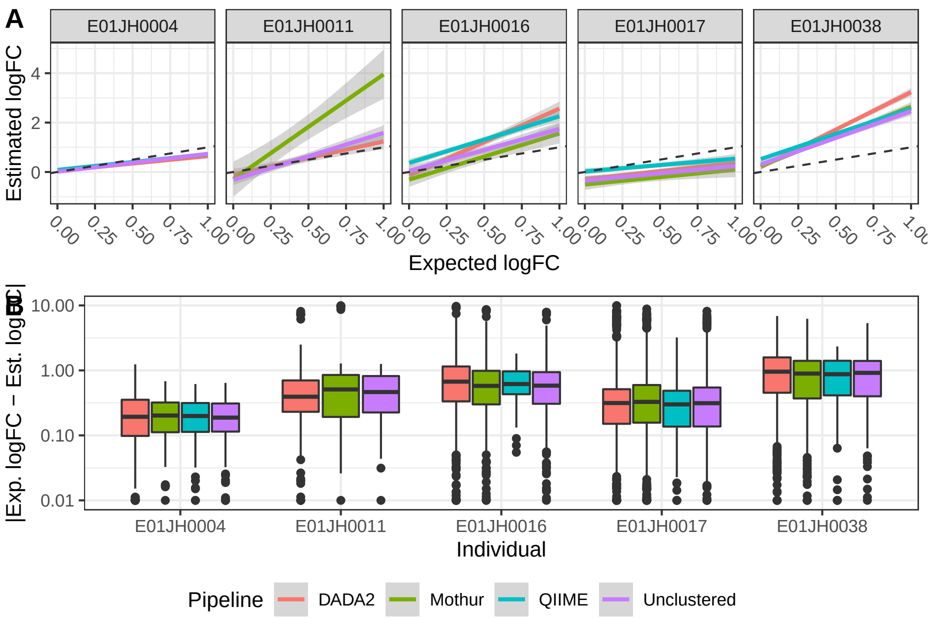

Figure 10: Differential abundance quantitative assessment for un-moderated edgeR logFC estimates, i.e. without using a prior count offset. (A) Linear model of the relationship between estimated and expected log fold-change relative abundance between titrations for PRE-specific and PRE-dominant features by pipeline and individual, line color indicates pipelines. Dashed grey line indicates expected 1-to-1 relationship between the estimated and expected log fold-change. (B) Log fold-change error (|exp-est|) distribution by pipeline and individual.

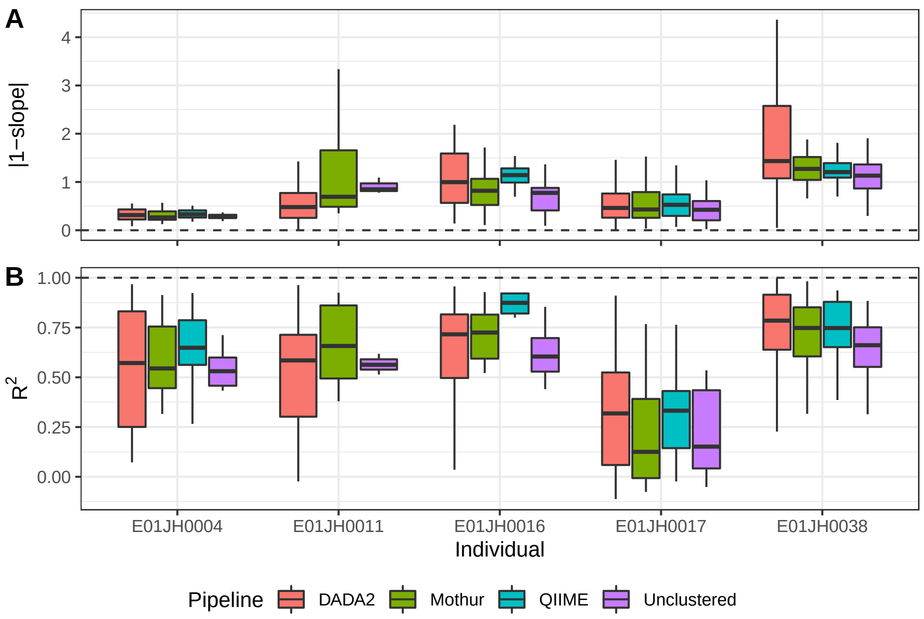

Figure 11: Feature-level differential abundance assessment for un-moderated edgeR logFC estimates, i.e. without using a prior count offset. Log-fold change error bias (A) and variance (B) metric distribution by subject and pipeline. The bias $(1 - slope)$ and variance (R^2) metrics are derived from the linear model fit to the estimated and expected log fold-change values for individual features. Boxplot outliers, $1.5 \times IQR$ from the median were excluded from the figure to prevent extreme metric values from obscuring metric value visual comparisons.
