# Supplementary material for: A framework for assessing 16S rRNA marker-gene survey data analysis methods using mixtures
Source: Microbiome. 2020 Mar 13;8:35. doi: 10.1186/s40168-020-00812-1 (PMC7071580; doi:10.1186/s40168-020-00812-1)
Supplement: Supplementary file 2 — Additional file 2 Sample ID Error and Correction. [file 40168_2020_812_MOESM2_ESM.docx]

**Sample ID Error and Correction**

While scientist strive for perfection, we are all human and mistakes happen. It is important to identify and correct errors as soon as possible so that they do not perpetuate in the scientific literature. During peer-review we discovered an error in our sample labels thanks to an additional figure requested by one of the reviewers. With proper documentation of the sample layout we confirmed and corrected the error. We are grateful to the keen reviewers for suggesting the additional figure that allowed us to identify and correct the error prior to publication. Here we describe the error, it’s identification, and correction. We hope that the lessons learned here, the importance of appropriate safeguards for preventing and recovering from such errors will help other scientists prevent, identify, and correct similar errors.

We described our initial mixture and sequencing experimental design in a study protocol document. We developed the initial protocol to minimize the impact of potential sample cross-contamination and propagation of pipetting biases (Supplemental Fig. 1A). But the execution of the protocol was not practical. To minimize pipetting errors in the laboratory, laboratorian setup and execute protocols in a systematic manner. Therefore, we revised the protocol based on feedback from the co-authors performing the laboratory experiments. Unfortunately, the sample sheet was not appropriately updated to reflect these changes. Due to the diligent documentation of the laboratory procedure, we have photographic evidence of the actual sample layout (Supplemental Fig 1B).

­­­


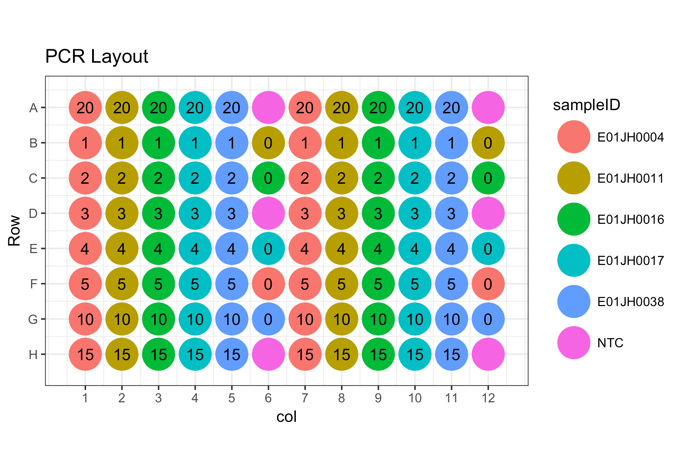

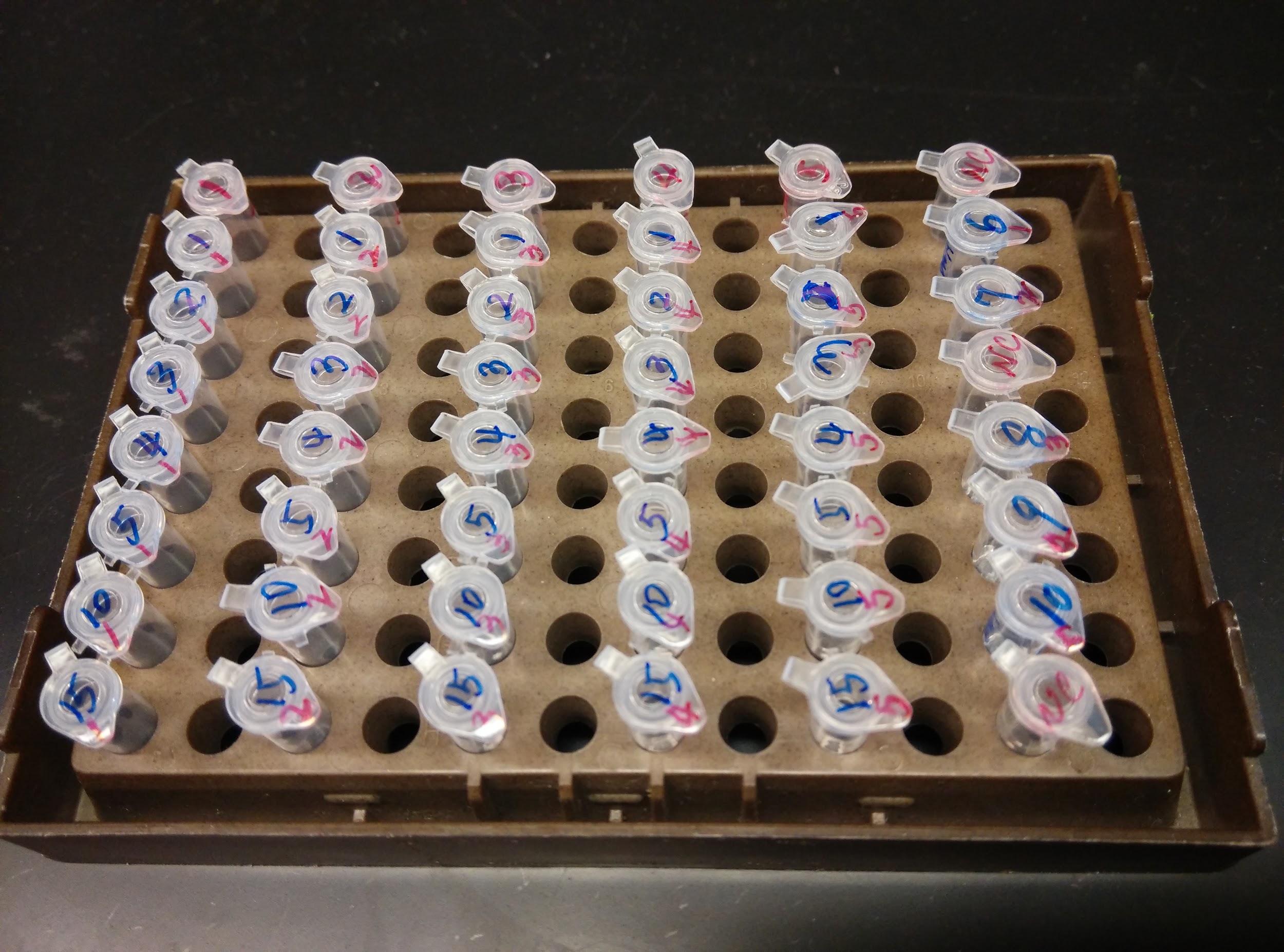


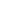

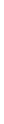

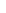

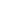

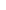

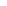

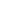

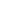

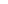

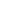

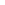

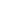

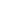

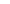

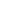

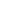

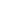

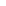

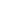

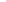

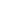

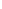

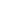

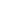

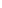

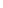

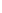

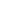


Figure 1 Titration series (A) experimental design plate layout and (B) image of the titration series plate layout for the titration series taken after sample mixing and prior to the initial 16S rRNA PCR.

We identified the sample sheet error while generating an additional figure requested by of the manuscript reviewers. The reviewer requested a PCA analysis of all the samples and titrations to show that the titrations were behaving as expected (Supplemental Fig. 2A).
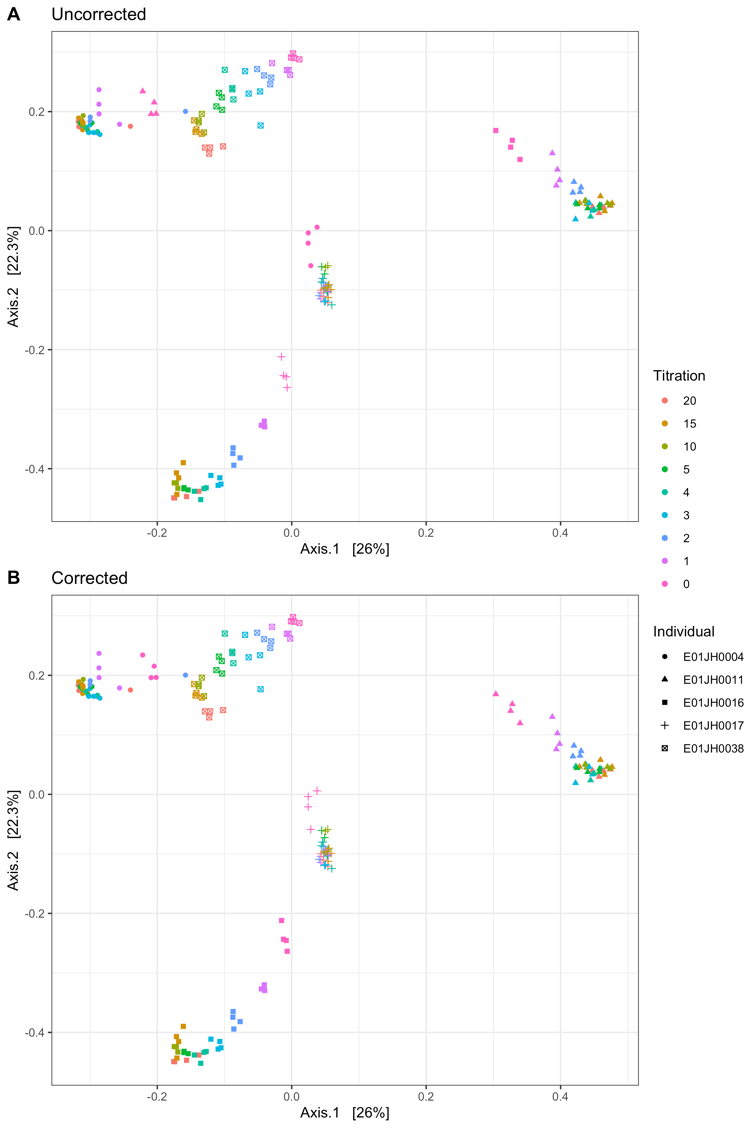
 We generated similar figures during our initial exploratory data analysis but only on individual samples and the expected titration trends were observed. But when we plotted the titration series for all five individuals on one plot we identified the potential error, the unmixed post-exposure samples were grouping incorrectly for four of the five individuals. Based on the plot alone we hypothesized that we assigned the unmixed post-exposure samples to the wrong individuals. We then went back and looked at the plate map picture taken in the lab prior to the initial 16S rRNA PCR (Supplemental Fig. 1B). The sample ids on the tubes in the image were consistent with our hypothesized sample ids. We corrected the sample ids and re-ran the PCA analysis resulting in the expected trend with samples grouping by individual and titration (Supplemental Fig. 2B).

There are a number of lessons learned with regards to preventing, identifying, and correcting similar sample sheet errors. A key factor in the prevention and correction of sample sheet error is extensive documentation of the work that was done in the laboratory, specifically the sample plate layout. As a general rule, words are good, but photographs are great. The sample sheet error could have been prevented by initially verifying that the sample sheet used in the data analysis was consistent with the experimental sample layout by generate the sample sheet with the laboratory image and not the experimental design. The sample sheet error could have also been identified as part of the initial exploratory data analysis by inclusion of figures specifically for sample sheet validation. Identification of appropriate statistical methods and figures to test a hypothesis is usually considered as part of the experimental design. But researchers rarely consider statistical methods and data visualizations for validating sample labels. By considering sample label validation during the experimental design phase of the of the study researchers can easily identify and correct these errors during the initial data analysis.

It is our hope that by documenting the sample sheet error identification and correction that occurred as part of this study that others can learn from our experience to help prevent them from making similar mistakes but also to know that these things happen in science and to prevent mistakes from perpetuating in the scientific literature it is important to consider preventative measures as part of the initial study design.
